# Epilepsy lesion localization method based on brain function network

**DOI:** 10.3389/fnhum.2024.1431153

**Published:** 2024-07-10

**Authors:** Chunying Fang, Xingyu Li, Meng Na, Wenhao Jiang, Yuankun He, Aowei Wei, Jie Huang, Ming Zhou

**Affiliations:** ^1^School of Computer and Information Engineering, Heilongjiang University of Science and Technology, Harbin, China; ^2^Department of Neurosurgery, The First Hospital of Harbin Medical University, Harbin, China; ^3^Faculty of Computing, Harbin Institute of Technology, Harbin, China

**Keywords:** SEEG, brain network, ENCS, persistent homotopy, SOZ

## Abstract

**Objective:**

In the past, the localization of seizure onset zone (SOZ) primarily relied on traditional EEG signal analysis methods. However, due to their limited spatial and temporal resolution, accurately pinpointing neural activity was challenging, thereby restricting their clinical applicability. Compared with traditional EEG signals, SEEG signals have superior spatial and temporal resolution, and can more accurately record neural activity near epileptic foci, making them better suited for studying SOZ. In addition, the traditional EEG signal analysis methods still have limitations, mainly focusing on the analysis of local signal features, while ignoring the complexity and interconnection of the overall brain network. How to more accurately locate SOZ is still not well resolved. The purpose of this study is to develop an effective positioning method for more accurate positioning.

**Method:**

To overcome these limitations, this study proposed a model integrating brain functional network analysis with nonlinear dynamics. We utilized weighted phase lag index (WPLI) to construct brain functional network, epilepic network connectivity strength (ENCS) as the feature, and introduced persistence entropy (PE) for feature fusion, subsequently employing support vector machine (SVM) classification.

**Results:**

The proposed method was verified on the HUP-iEEG dataset, our solution identified the SOZ with 0.9440 accuracy, 0.9848 precision, 0.8974 recall rate, 0.9340 *F*1 score and 0.9697 area under the ROC curve across patients, which outperforms the existing approaches. It exhibits a 2.30 percentage point enhancement in localisation accuracy along with a 2.97 percentage points in AUC compared to others.

**Conclusion:**

Our method consider the interactions between nodes in brain network connections, as well as the inherent nonlinear and non-stationary properties of neural signals, to be more robust.

## 1 Introduction

Epilepsy is a chronic neurological disorder caused by abnormal discharges of neurons in the brain, now ranking as the second most common neurological condition globally. During a seizure, electrical activity in the brain is disrupted, allowing for dysfunction and impaired communication between areas of the brain, which leads to a number of temporary symptoms including loss of consciousness, staring spells, and movement disorders. According to the World Health Organisation, approximately 5 million people worldwide are diagnosed with epilepsy annually. In high-income countries, an estimated 49 out of every 100,000 people are diagnosed with epilepsy each year. In low-and middle-income countries, this number may be as high as 139 per 100,000 people diagnosed with epilepsy, so nearly 80% of epilepsy cases occurring in these regions. Among epilepsy patients, 20–30% suffer from medically refractory epilepsy, whose resistance to conventional antiepileptic medications makes it difficult to achieve significant results with conventional treatment (Kharibegashvili, 2020). In this challenging context, surgery has emerged as a potential treatment to explore, particularly through resection of the suspected epileptogenic zone (EZ), the specific brain region that triggers seizures. The idea behind surgical removal of EZ is to eliminate seizures by pinpointing and removing areas of abnormal activity in the brain. However, this procedure may involve an impact on normal brain tissue, especially if the EZ is adjacent to a functionally important brain region. This can lead to surgical risks and potential neurological damage. Even after surgical removal of the epileptogenic zone, there is still a risk of seizure recurrence. This may occur due to the surgery failing to completely remove the epileptogenic tissue, or other brain regions being activated. To better understand these complexities, researchers employ various neuroimaging techniques, such as functional magnetic resonance imaging (fMRI), electroencephalography (EEG), stereo electroencephalography (SEEG), and positron emission tomography (PET), to study patterns of brain activity and connectivity to unravel the complex processes involved in the propagation of epilepsy.

SEEG is a technique employed to record electrical signals in epilepsy or other neurological disorders (Gao et al., 2018). By implanting multiple electrodes into the patient’s brain, it can capture electrical signals from deeper brain regions, aiding doctors to determine the location and extent of seizure onset points. Additionally, SEEG can also furnish more precise information about brain function location prior to surgical intervention, thus preventing unnecessary damage to normal brain tissue. Compared with traditional intracranial EEG, SEEG boasts superior spatial resolution, precision, safety, and controllability (Pei et al., 2021). Consequently, SEEG stands out as a powerful tool for conducting accurate epilepsy brain electrical signal analysis. In recent years, mounting studies have shown that epilepsy is not only a static brain dysfunction, but also a complex pathophysiological process involving dynamic brain networks (Sun et al., 2010). Therefore, utilizing SEEG as an brain electrical signal acquisition technology and using a dynamic brain network analysis method for epileptic disorders is of great significance for the localisation of seizure onset zone (SOZ).

Currently, domestic and international research on SOZ localisation have focused on the use of high frequency oscillations (HFO) rates, functional connectivity and graph theory, phase-amplitude coupling, or hybrid methods, with fewer studies on SOZ localisation using brain networks. For instance, Wu et al. (2021) utilized K-centre clustering to identify HFOs and used the detected HFO concentrations to localise SOZ. A. Nazar and Sanjay (2023) automated the discovery of HFOs from multichannel intracranial EEG signals, which led to epileptic zone localisation by calculating the HFO rate/min. Similarly, Sadek et al. (2023) enhanced and automated HFO localisation by developing unsupervised clustering methods based on the analysis tool to improve and automate HFO detection, used a CNN feature extractor to finally localise seizure regions. Grattarola et al. (2022) used correlation and phase-locked values to quantify the coupling between different brain regions and extracted FN metrics to perform epileptic focal zone detection by training a GNN. Jiang et al. (2022) estimated the directional connectivity through the information flow and inferred the excitation-inhibition ratio from 1/f power slopes, and later combined a balanced random forest model with resting state connectivity to localise epileptic focal areas. It is worth noting that although these methods have made useful attempts to localise SOZ, we must recognise that there are still some obvious shortcomings and limitations of the existing methods. Primarily, these methods usually focus on localised signal features, neglecting the complexity of brain networks. Epilepsy, as a network disease, involves complex interactions among multiple brain regions. Focusing solely on specific signals or changes in individual channels may not provide a comprehensive understanding of the disease mechanism, thereby affecting the accuracy and comprehensiveness of lesion localisation. Additionally, the features used for localisation, such as HFO rate, are inherently limited since HFO do not always occur in the early stages of seizures and not all epileptic patients exhibit significant HFO, which makes it possible that, in some cases, these methods may overlook the presence of epileptic foci or lead to inaccurate localisation. Therefore, this paper proposes the utilization of a weighted phase lag index (WPLI) to construct a functional brain network from the perspective of phase synchronisation in order to locate epileptogenic zone more comprehensively and accurately. This approach is anticipated to overcome the shortcomings of existing methods and better reflect the nature of epilepsy as a network-based disease, thereby providing a more feasible way to improve the treatment of epilepsy patients. In addition, HFO and phase-amplitude coupling methods are usually based on certain modelling assumptions, such as signal linearity and stability. However, the neural activities of the human brain reflected in SEEG are complex activities characterised by nonlinear dynamics, and the neural signals may exhibit nonlinear and nonstationary properties in epileptic states, leading to the violation of model assumptions. Therefore, in this paper, we will leverage the nonlinear dynamics features to further elucidate the dynamic behaviours of the lesion area, and integrate the brain functional network features with nonlinear dynamics features to establish a comprehensive localisation model that takes into account the patient’s brain functional connectivity patterns and dynamic behavioural features, thereby enhancing the accuracy and reliability of the localisation of SOZ. With this approach, we expect to provide epilepsy patients with more precise diagnosis and treatment plans, ultimately improving their quality of life.

The second section of this paper describes the dataset used in this paper and the methodology applied in the experiments. The third section describes the experimental procedure and the results obtained from the experiments. The fourth section discusses the innovations of this paper. The fifth section summarises the findings of this paper, and the final section briefly describes the shortcomings of this paper and the directions for future research.

## 2 Materials and methods

**Figure 1:**
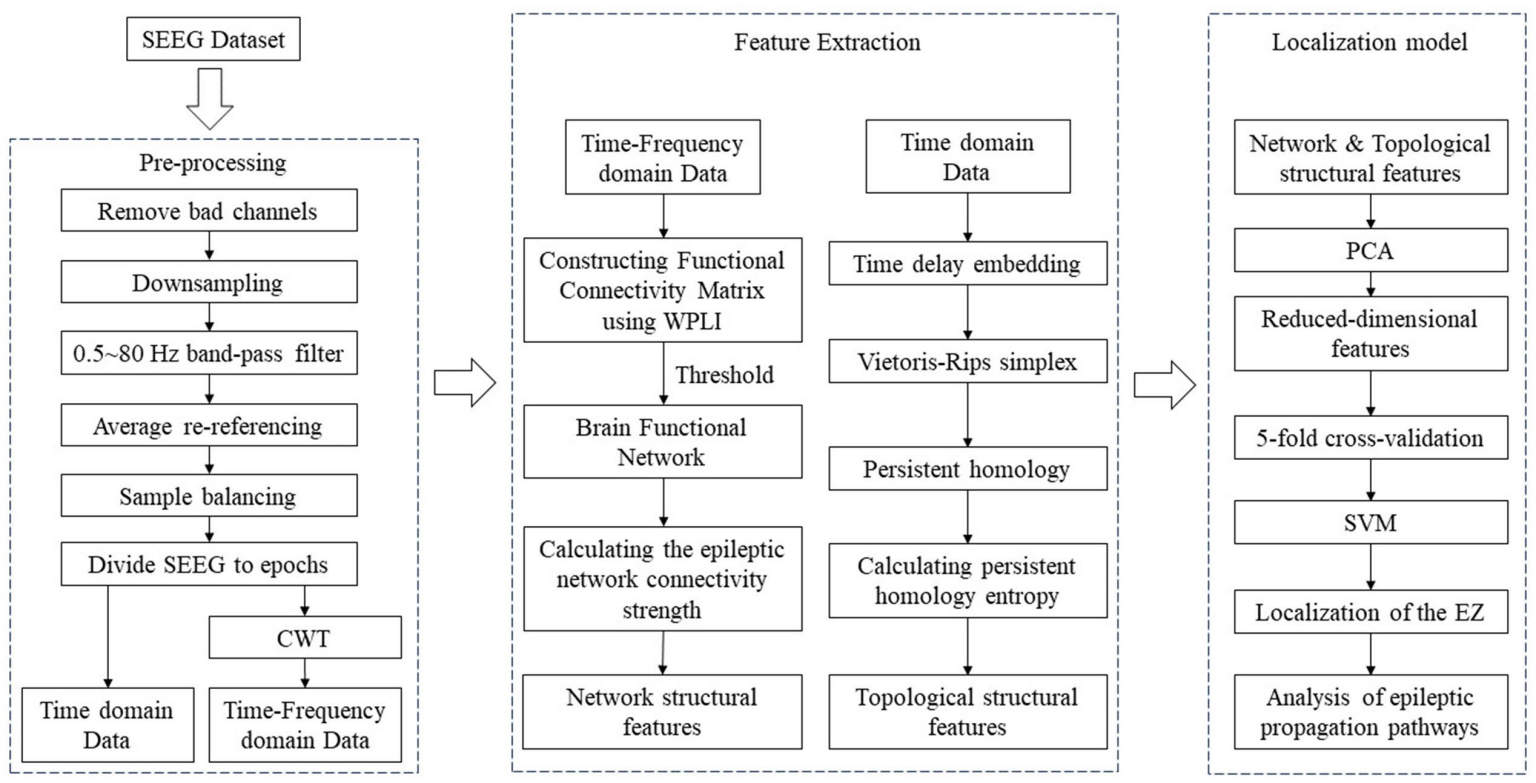
The flowchart of the algorithm.

In this study, we analysed SEEG data from 22 epilepsy patients. The data were pre-processed and then WPLI was applied to calculate network connectivity from phase and time-frequency perspectives. Dynamic thresholds were set such that each functional brain network graph contained multiple brain nodes with high connectivity values. Epileptic network connectivity strength (ENCS) was utilized to identify and visualise significant nodes for functional connectivity. In addition, SEEG data from 22 epilepsy patients were time delay embedded and transformed into point cloud representations in 3D space. Subsequently, persistent homology computation was performed using Vietoris–Rips simplex complex shapes to obtain their 3D persistent homology features. In this process, the persistent homology entropy was employed as a feature to characterize the dynamic properties of SEEG data, so as to facilitate a deeper exploration of the complex dynamic properties of SEEG signals. Finally, the network structural features and topological structural features were utilized for the localisation of SOZ and the analysis of propagation pathways. The flowchart of the algorithm is illustrated in Figure 1, depicting specific steps including raw data pre-processing, calculation and extraction of network structural and topological features, localization of the SOZ and analysis of epileptic propagation pathways.

### 2.1 Dataset

The experimental dataset used in this paper was sourced from the Hospital of the University of Pennsylvania, which can be accessed at the following link: 10.18112/openneuro.ds004100.v1.1.3. It comprised intracranial electro-encephalogram (iEEG) signal data from 55 patients, consisting of 28 males and 27 females, all diagnosed with refractory epilepsy and who had undergone surgical procedures. Among these patients, 36 were treated with electrode implantation using SEEG and 19 patients received electrode implantation using cortical electro-encephalography (EcoG), with the number of electrodes implanted varied depending on the patient’s condition. The dataset also include information about the patient’s handedness, the type of treatment administered, the surgical target point, the lesion status on MRI, and surgical outcomes with or without seizures. For patients undergoing SEEG electrode implantation, the dataset includes 10 min interictal data, and data for seizure periods of varying duration.

During the selection of experimental data, this paper chose (1) electrode implantation mode as SEEG, and (2) post-surgical outcome as S, i.e., successful surgery and no seizure. (3) No interictal epileptiform discharges (IEDs). In this way, the data of 22 patients meeting the experimental criteria were screened out, as shown in Table 1.

**Table 1:** Patient information.

Patient ID	Age (y)	Sex	Hand	Implant	Target	Therapy	Outcome
sub-HUP117	39	M	L	SEEG	Temporal	Resection	S
sub-HUP130	46	F	L	SEEG	MFL	Ablation	S
sub-HUP133	52	F	L	SEEG	MTL	Ablation	S
sub-HUP138	38	M	L	SEEG	MTL	Ablation	S
sub-HUP142	30	M	L	SEEG	MTL	Ablation	S
sub-HUP144	31	M	R	SEEG	Temporal	Resection	S
sub-HUP150	17	M	R	SEEG	Insular	Ablation	S
sub-HUP151	33	M	R	SEEG	MFL	Ablation	S
sub-HUP157	25	M	L	SEEG	MTL	Ablation	S
sub-HUP160	45	F	R	SEEG	Temporal	Resection	S
sub-HUP162	35	F	L	SEEG	MTL	Ablation	S
sub-HUP164	34	F	L	SEEG	MTL	Ablation	S
sub-HUP166	26	M	L	SEEG	Temporal	Resection	S
sub-HUP171	50	M	L	SEEG	Frontal	Ablation	S
sub-HUP172	28	F	L	SEEG	Frontal	Ablation	S
sub-HUP173	24	F	R	SEEG	Temporal	Resection	S
sub-HUP180	28	F	L	SEEG	Frontal	Ablation	S
sub-HUP181	31	F	L	SEEG	Temporal	Ablation	S
sub-HUP185	38	M	L	SEEG	MTL	Ablation	S
sub-HUP187	25	M	R	SEEG	MTL	Ablation	S
sub-HUP188	24	F	L	SEEG	Frontal	Resection	S
sub-HUP190	25	M	L	SEEG	MTL	Resection	S

Sex, F stands for female, M stands for male; Hand, R for right-handed, L for left-handed; Outcome, S stands for successful surgery without seizures, F stands for surgical failure and seizures still recurring after surgery.

### 2.2 Data preprocessing

During the pre-processing stage, the MATLAB R2020a software and the EEGLAB toolbox were employed. Initially, the bad channels marked in the dataset were removed and the sampling frequency was reduced to 200 Hz to diminish redundancy while preserving periodic information. Subsequently, due to the 60 Hz industrial frequency interference affecting SEEG signals caused by external power supply and circuitry during acquisition, a 60 Hz trap filter was utilized to eliminate power supply noise, thus enhancing the quality of the SEEG signals, and making the subsequent analyses and researches more reliable. Furthermore, we utilized a bandpass filter with a cutoff frequency of 0.5 Hz to 80 Hz, aiming to concentrate our analysis on the specific signal band relevant to our interest. In order to eliminate the effect of original referencing on the recorded data, common average re-referencing was performed in the preprocessed data.

As the proportion of SOZ electrodes is small compared to all electrodes implanted in each patient in the experimental dataset, resulting in sample imbalance, which is not conducive to the later localizing of the SOZ, adjustments need to be made to balance and segment the samples. Taking sub-HUP142 as an example, the SEEG data of this patient consists of 116 channels, among which 8 channels are bad channels, and after removing the bad channels, there remain 108 channels, with 16 channels belonging to the SOZ, and 92 channels belonging to the non-SOZ, resulting in highly unbalanced samples. In this paper, we adopt a window length of 30 s and a window shift of 25 s for the non-SOZ channels to perform the slicing, while the window length of 30 s and 25 s for the SOZ channels to perform the slicing. Subsequently, one SEEG slice from a non-focal area channel and five SEEG slices from non-focal area channels are combined in chronological order to form a new full-channel SEEG dataset, effectively increasing the number of samples for the focal area channels fivefold. After adjusting the balance of the samples, the total number of channels (after removing the bad channels) becomes 172, with the number of focal area channels reaching 80. As shown in Supplementary Figure S1.

Finally, the processed SEEG signals were divided into five different frequency bands: delta band (1 Hz–3 Hz), theta band (3 Hz–7 Hz), alpha band (7 Hz–13 Hz), beta band (13 Hz–30 Hz), and gamma band (30 Hz–60 Hz). Further analysis was conducted on the epileptic EEG signal data across these different frequency bands.

### 2.3 Brain function network

Functional connection refers to the interactions between different brain regions or neurons, which are manifested in some degree of concerted activity or synchronisation. Such connections reflect patterns of information transfer in the brain and are essential for understanding brain function and studying the workings of the nervous system. In neuroscience research, a range of metrics is often used to quantify the properties of these functional connections. Among them, mutual information (Pluim et al., 2003), Pearson’s correlation coefficient (Antony et al., 2013), phase-locked value (PLV) (Lachaux et al., 1999), and phase-locked index (PLI) (Stam et al., 2007) are metrics commonly used to measure the phase synchronisation of brain signals. To reveal the phase relationship between brain regions more accurately, we will construct a brain functional network using WPLI. After calculating the functional connectivity index values between every two electrodes in each frequency band of each time window, a two-dimensional matrix can be obtained for each frequency band of each time window. In this matrix, the electrode points serve as nodes, and the functional connectivity index values are used as edges connecting the nodes, thereby constituting a dynamic brain functional network. Subsequently, a brain network sparsity of 40–50% is adopted as the binarisation threshold. When the connection value between two nodes in the brain functional network exceeds the determined threshold, the connection between the two nodes is considered to exist as an edge, with the edge value set to 1, otherwise it is set to 0. In this way, the functional network is binarised, and the constructed brain functional network is transformed into a binary matrix network for the subsequent calculations individual feature attributes of the brain functional network for feature extraction.

Weighted phase lag index (WPLI), a refinement of the phase lag index, incorporates weighting of the lag magnitude to mitigate the influence of small phase differences (Vinck et al., 2011). Compared with the traditional phase synchronisation index, WPLI improves the robustness, enabling more accurate reflection of the strength and stability of phase synchronization. Consequently, WPLI facilitates the comprehensive exploration of functional connectivity patterns among the brain regions surrounding the epileptic focal area, thereby enhancing our understanding of interactions between the SOZ and other brain regions. Benefitting from the high temporal and spatial resolution information provided by WPLI, it becomes feasible to discern the phase synchronisation patterns across different frequency bands between brain regions. This capability enables precise identification of abnormal connectivity patterns around epileptic focal regions and furnishes more specific localization information.

The calculation of the WPLI between electrode X and electrode Y in a specific frequency band within a certain epoch involves several steps after preprocessing. Firstly, the preprocessed EEG signal data from electrode X and electrode Y in each epoch and the specific frequency band are subjected to the Hilbert Transform. Next, the cross-spectrum of the resolved signals of electrode X and electrode Y in that frequency band should be calculated. Finally, the WPLI between electrode X and electrode Y in that frequency band is obtained.

Where WPLI is calculated as shown in Eq. 1:


(1) $$\mathrm{WPLI}=\frac{\left|\overset{n}{\underset{i=1}{\sum }}I\left[C_{i}\left(t,f\right)\right]\right|}{\overset{n}{\underset{i=1}{\sum }}\left|I\left[C_{i}\left(t,f\right)\right]\right|}=\frac{\left|\overset{n}{\underset{i=1}{\sum }}I\left[X_{i}\left(t,f\right){Y_{i}}^{*}\left(t,f\right)\right]\right|}{\overset{n}{\underset{i=1}{\sum }}\left|I\left[X_{i}\left(t,f\right){Y_{i}}^{*}\left(t,f\right)\right]\right|}$$

where, $I\left(\text{.}\right)$ represents taking the imaginary part; $C_{i}\left(t,f\right)$ is the cross-spectrum between the data $X_{i}\left(t,f\right)$ and $Y_{i}\left(t,\;f\right)$ of the *i*th experiment; $X_{i}\left(t,f\right)$ and are the results of the Hilbert transforms of the data $X_{i}\left(T\right)$ and $Y_{i}\left(T\right)$ of the *i*th experiment, respectively; and ${Y_{i}}^{*}\left(t,f\right)$ denotes the complex conjugate of $Y_{i}\left(t,f\right)$ .

The value of WPLI ranges from 0 to 1, where 0 represents no correlation, indicating no phase difference between the two signals, and 1 represents the maximum correlation, indicating that the two signals are completely phase-locked. The higher the value of WPLI, the higher degree of the phase synchronisation between the two signal data.

### 2.4 Epilepic network connectivity strength

In functional brain network graphs, epilepic network connectivity strength (ENCS) serves as a vital metric for quantifying the extent of phase-synchronized connectivity among nodes. ENCS represents the count of phase-synchronous connections between a node and other nodes, signifying the significance and influence of the node within the network. The calculation of ENCS can be achieved by tallying the number of edges between a node and other nodes, i.e., tallying the number of neighbouring nodes of the node in the graph, as shown in Eq. 2.


(2) $$k_{i}=\underset{j\in G}{\sum }a_{ij}$$

where *G* denotes the constructed functional brain network, and *a_ij_* denotes the value of column *j* of row *i* of the binary matrix network of the functional brain network. A higher ENCS means that there is more phase synchronisation between the node and other nodes, reflecting the node’s important position in the network. ENCS can provide information about the connection strength of nodes and the overall network topology, which provides valuable guidance for epileptic focal area localisation and brain network analysis.

### 2.5 Persistent homotopy

Persistent homotopy is a mathematical tool used for analysing the topology of a dataset, which extracts crucial information by considering changes in the topology of the dataset across various scales. Persistent complex, the foundation of persistent homotopy, is a method for transforming a dataset into a series of simplexes. Each simplex represents the topology of the dataset at a particular scale. By creating persistent complexes and employing persistent homotopy, we can extract persistence features from the dataset. These features include connected components, holes, voids, and more, allowing us to analyze their persistence across different scales. This process reveals the underlying topological characteristics of the dataset.

#### 2.5.1 Delayed embedding to construct point clouds

Persistent homology methods typically assume point cloud as input data. However, SEEG data are in the form of time series and are not directly applicable to persistent homotopy analysis. Therefore, in order to analyse EEG signal data within a persistent homotopy framework, it must first be converted into point cloud format. This conversion is usually achieved by using various embedding methods. In this study, we employ a dynamic spatial reconstruction technique, which utilizes the delayed embedding theorem proposed by Takens (2006) to map the 2D time series embedding of a single channel into the corresponding high-dimensional phase space, thereby obtaining a data representation in point cloud form. The core principle of this technique is to leverage the time series data’s delay information to construct a high-dimensional phase space representation, revealing the data’s dynamical structure and nonlinear properties.

Suppose there is a one-dimensional time series data *x*(*t*), where *t* denotes the time. Takens delayed embedding theorem asserts that, for a dynamical system, its evolution should be continuous in phase space, i.e., the states at the neighbouring time points are also close in phase space. Therefore, by reconstructing the time series data with a certain time delay (lag), a high-dimensional phase space representation can be obtained, which can better describe the dynamics of the system. Specifically, as shown in Eq. 3, given a time series data *x*(*t*):


(3) $$X=\left[x\left(i\right),\;x\left(i+\tau \right),\;x\left(i+2\tau \right),\cdots ,\;x\left(i+\left(m-1\right)\tau \right)\right]$$

where *m* is the embedding dimension, *τ* is the time delay. By selecting an appropriate values for *m* and *τ*, the reconstructed phase space can accurately capture the dynamics of the original time series data. The most commonly used methods for determining these parameters are the average mutual information (AMI) method for selecting the optimal delay time, and the false nearest neighbour (FNN) algorithm for selecting the optimal dimension (Wallot and Mønster, 2018).

#### 2.5.2 Tectonic simplex complex form

In topological spaces, the continuous construction of simplex complexes for point clouds is essential to describe and study high-dimensional topologies. A simplex complex is a topology consisting of simplexes, which are geometric objects in Euclidean space consisting of a set of vertices and all their possible combinations. Specifically, a *k*-dimensional simplex is a convex hull of *k* + 1 non-collinear points, which is a geometric shape encompassing all possible subsets such as vertices, edges, triangles etc. In a simplex complex, the structure of a topological space is constructed by assembling simplexes and their boundaries. It contains a series of simplexes and all their possible combinations of faces, bodies, etc. These combinations form the topological structure of the complex, elucidating the connectivity relations and morphology in space. In this paper, we utilize the construction of Vietoris–Rips complex (Bauer, 2021) to establish a simplex complex for point cloud datasets, which converts the original point cloud data into a topological structure in order to obtain the topological features of the point cloud data in the topological space for topological data analysis.

In the construction of the Rips complex, a scale parameter *r* is provided. The structure of the Rips complex encompasses all subsets of the point set *X* with a diameter no greater than *r*. This implies that for any point set *X* with a diameter at most *r*, the Rips complex can be established as a topology. The containment relationship among these simplexes aligns with the containment relationship among the corresponding subsets, as shown in Eq. 4. This construction captures the local geometry of the dataset *X* and facilitates the analysis of its topological features and structure of the data.


(4) $$K\left(r\right)=\left\{\sigma \subseteq X|\dim\left(\sigma \right)\leq r\right\}$$

where dim(*σ*) = *σ* − 1 denotes the dimension of the simplex *σ*, and the d-skeleton of a simplex complex comprises all simplexes in the simplex complex *K* with dimension not exceeding *d*. As *r* increases, the topology of the complex undergoes changes, gradually transitioning from simple to complex. This gradual augmentation of simplexes can be visualised as a progressively expanding filter (Edelsbrunner and Harer, 2022), unveiling the multiscale topology of the dataset. This can be expressed as:

$$\emptyset \subseteq K_{0}\subseteq K_{1}\subseteq \cdots \subseteq K_{n-1}\subseteq K_{n}=K$$

#### 2.5.3 Persistent homology analysis

After constructing a simplex complex, it is imperative to unveil the topology of the space by computing the homotopy groups of the simplex complex. In this study, we focus solely on the zero-dimensional, one-dimensional and two-dimensional homotopy groups. By analysing the persistence of the homotopy groups, we can identify persistent topological structures in the space such as holes, rings, etc., thereby enhancing our comprehension of the topological characteristics of the space. Subsequently, the persistence of the homotopy groups is analyzed by tracking the filtration at the moment of creation (i.e., birth), the filtration at the moment of termination (i.e., death), and the dimensionality of each homology class. This parameters can be visualised represented using persistence graphs. A persistence graph is a two-dimensional graph where the *x*-axis represents the moment of birth and the *y*-axis represents the moment of death. For each homotopy class that is born at *i* and dies at *j* during its lifespan, a point is plotted at coordinates (*i*, *j*) on the two-dimensional plane. This collection of points is often denoted as D = {(*b_1_*, *d_1_*), …, (*b_k_*, *d_k_*)}. where points further away from the diagonal line symbolize homotopy classes that persist longer, indicating more enduring structures. Within the persistence graph D, the persistence interval for each point *x* = (*b*, *d*) can be defined as |*b* − *d*|.

#### 2.5.4 Topological feature representation

Persistence diagram is a valuable tool for visualising the results of persistent homology, comprising a series of points, each representing a persistence interval that illustrates persistent features within a dataset and their duration (Chepushtanova et al., 2015). However, the aggregate structure of these points makes it challenging to apply traditional statistical concepts, such as mean or median, directly. This difficulty arises because these points are not continuous data, but represent the persistence of various topological features within the dataset. Despite the intuitive utility of persistence diagrams in grasping the characteristics of topological structures, their visual nature poses challenges for quantitative analysis using conventional statistical methods (Otter et al., 2017; Barnes et al., 2021; Pun et al., 2022).

Currently, five quantitative methods are commonly utilized in persistent homology analysis including homology class lifespans (Bendich et al., 2016), persistence landscapes (Bubenik, 2020), persistence silhouettes (Chazal et al., 2014), persistence images (Adams et al., 2017) and persistence entropy (Rucco et al., 2016). Among these methods, persistence entropy stands out for providing a perspective on the entire persistent homology graph in a quantitative manner, allowing for the measurement of the complexity of the persistent homology results. It integrates the distribution and diversity of persistence intervals, enabling a more comprehensive characterization of the topology in the dataset. In the calculation of persistence entropy, the definition of information entropy is usually adopted, i.e., the distribution of persistence intervals is treated as a probability distribution, and subsequently the entropy value is calculated using the entropy formula. As shown in Eq. 5.


(5) $$PE\left(D\right)=-\underset{i\in D}{\sum }\frac{p\left(i\right)}{𝓁\left(D\right)}\log_{2}\left(\frac{p\left(i\right)}{𝓁\left(D\right)}\right)$$

where PE (*D*) represents the value of persistence entropy; *p*(*i*) denotes the persistence interval of a single point; $𝓁\left(D\right)=\underset{i\in D}{\sum }p\left(i\right)$ is the sum of the persistence intervals of all points in the graph. A higher persistence entropy indicates that the distribution of persistence intervals is more irregular and diverse, reflecting the complexity of the topology in the dataset. A higher entropy value suggests the presence of more persistence features and topological changes in the dataset at different scales, whereas a lower entropy value indicates a relatively simpler topological structure.

### 2.6 Evaluation indicators

Indicators commonly used to evaluate the localisation of SOZ typically include the following:

Accuracy (AC) is a metric that quantifies the ratio of correctly located samples by the localisation method to the total number of samples. It serves as a fundamental evaluation criterion, indicating how accurately a localisation method locates the entire dataset. The higher the accuracy, the superior the performance of the localisation method. This is expressed in Eq. 6.


(6) $$\mathrm{AC}=\frac{TP+TN}{TP+TN+FP+FN}\times 100\%$$

where TP, true positive, represents the number of samples that were actually positive cases that were correctly positioned as positive cases; FN, false negative, represents the number of samples that were actually positive cases that were incorrectly positioned as negative cases; TN, true negative, represents the number of samples that were actually negative cases that were correctly positioned as negative cases; and FP, false positive, represents the number of incorrectly positioned positive cases in samples that were actually negative cases.

Precision (P), an indicator of the accuracy of positive category prediction, measures the proportion of correctly identified positive samples out of all samples classified as positive. It helps evaluate the model’s ability to avoid misclassifying negative category samples as positive. As depicted in Eq. 7.


(7) $$P=\frac{TP}{TP+FP}\times 100\%$$

Recall (R) evaluates the proportion of correctly identified positive samples out of all actual positive samples. It assesses the ability of the model to identify positive category samples and avoid misclassifying them as negative. The formula is presented in Eq. 8.


(8) $$R=\frac{TP}{TP+FN}\times 100\%$$

The *F*1 score, a harmonic mean of precision and recall, offers a balanced assessment of the performance of the model by considering both its accuracy and its capability to capture positive category samples. Eq. 9 presents the formula for calculating the *F*1 score.


(9) $$F1=\frac{2\times P\times R}{P+R}\times 100\%$$

## 3 Experiment and results

In this section, we outline the experimental and validation process employed in this study, comprising 4 main steps: construction and analysis of brain functional networks, characterisation of sustained homotopy topology, localisation of epileptic SOZ, analysis of epileptic propagation pathways. Each step is described in detail to provide a comprehensive understanding of the methodology used in this research. The process involves constructing a functional brain network based on the HUP-iEEG data, extracting dynamic features from the network, and conducting an analysis to assess the effectiveness and reliability of the extracted features. These steps are essential for investigating the operation of the brain during epileptic seizures and gaining deeper insights into the underlying mechanisms.

### 3.1 Construction and analysis of brain functional networks

During the brain functional network construction phase, WPLI was employed to construct the brain functional network for each sample data of each patient. This process yielded a two-dimensional matrix of *n* × *n* for each sample, where *n* denotes the number of channels after equalisation for that specific subject sample. Notably, this matrix exhibits completely symmetric along the main diagonal. Utilizing a brain network sparsity of a threshold, the functional network was binarised to eliminate weak connections, thereby mitigating the influence of these interferences on network analysis. This approach accentuates key connections with higher connectivity strengths, thereby enhancing the interpretability and visualization of the network.

There is no uniform consensus on how to select thresholds in brain network analysis. There are usually two main methods used to estimate thresholds: absolute thresholds and proportional thresholds. An absolute threshold is a specific connection strength value that indicates that only connections with strengths above that value will be retained in the network; the rest of the connections will be considered invalid and removed from the graph. The method of using absolute threshold is simple and straightforward, but it is susceptible to data size and specificity conditions. Therefore, when applied in different datasets, the absolute threshold may need to be adjusted to fit the characteristics of the data. Proportional thresholding, on the other hand, filters the connections in the network based on the percentage of connection strength. That is, the connections are sorted in descending order of connection strength and those with strength above a certain percentage are selected as valid connections. Compared with absolute thresholding, proportional thresholding is more flexible and stable because it can adapt itself to the size and characteristics of different datasets and maintain the relative sparsity and connectivity of the network.

**Figure 2:**
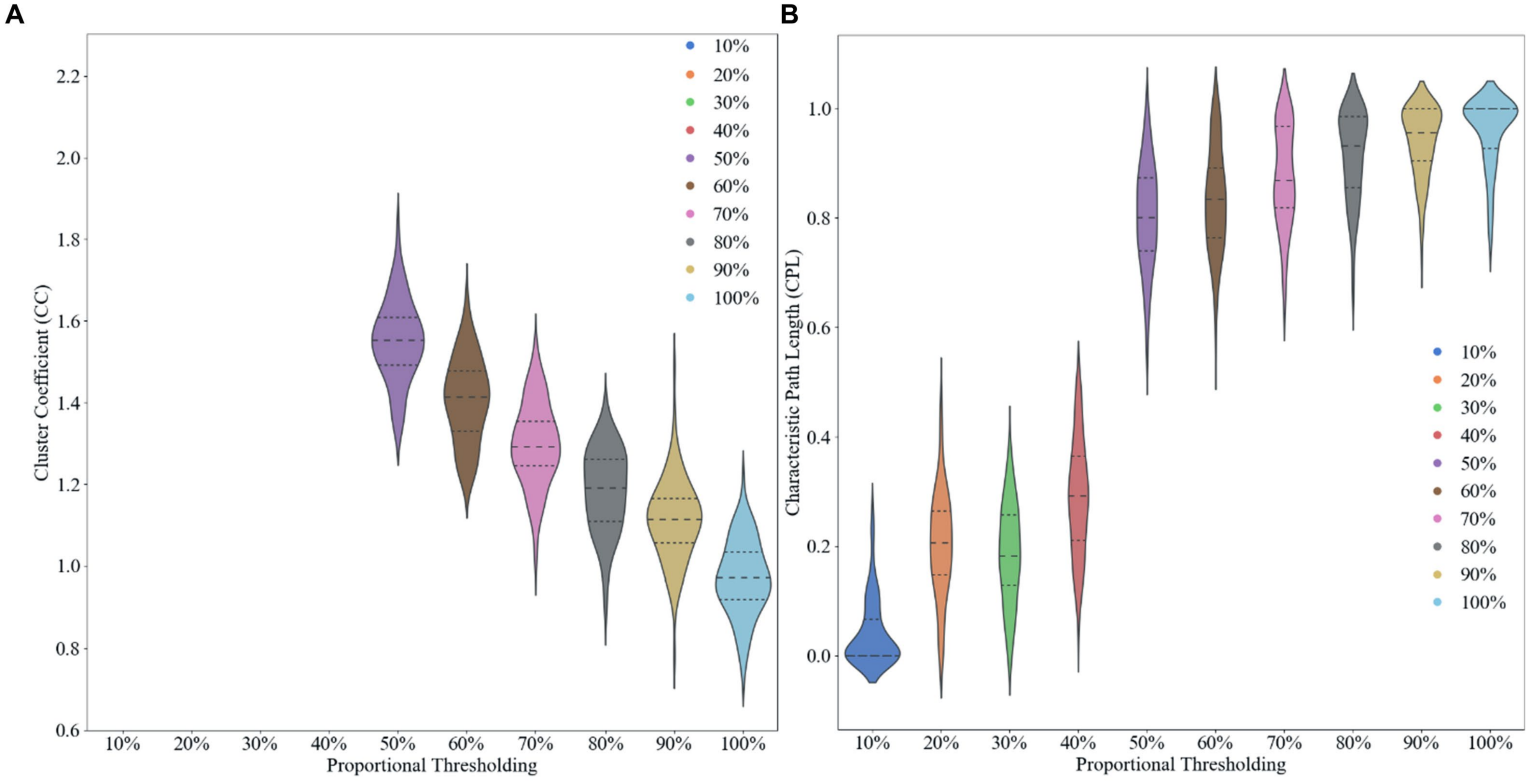
Effect of different proportional thresholds on graph metrics. **(A)** Clustering coefficient. **(B)** Characteristic path length.

We use proportional thresholding in our experiments to compute and analyze the characteristics of brain networks by sliding the thresholds with different sparsities (from 10 to 100% in steps of 0.1). We calculated the clustering coefficients and characteristic path lengths of the network at different thresholds after computing the functional connectivity matrix. Where the clustering coefficient reflects the degree of aggregation of nodes in the network, i.e., whether the neighbors of nodes tend to form close connections with each other. The characteristic path length, on the other hand, measures the average shortest path length between nodes in the network. By analyzing these metrics, we are able to determine an optimal threshold 50% that allows the network to maximize the sparsity of the network while preserving the small-world property, it can found in Figure 2. Choosing an appropriate threshold is critical to accurately reflect the functional structure of the brain network. Using a proportional threshold approach, we can ensure the stability and reliability of brain network properties at different sparsities, leading to a more comprehensive understanding of the connectivity of the brain’s functional networks.

**Figure 3:**
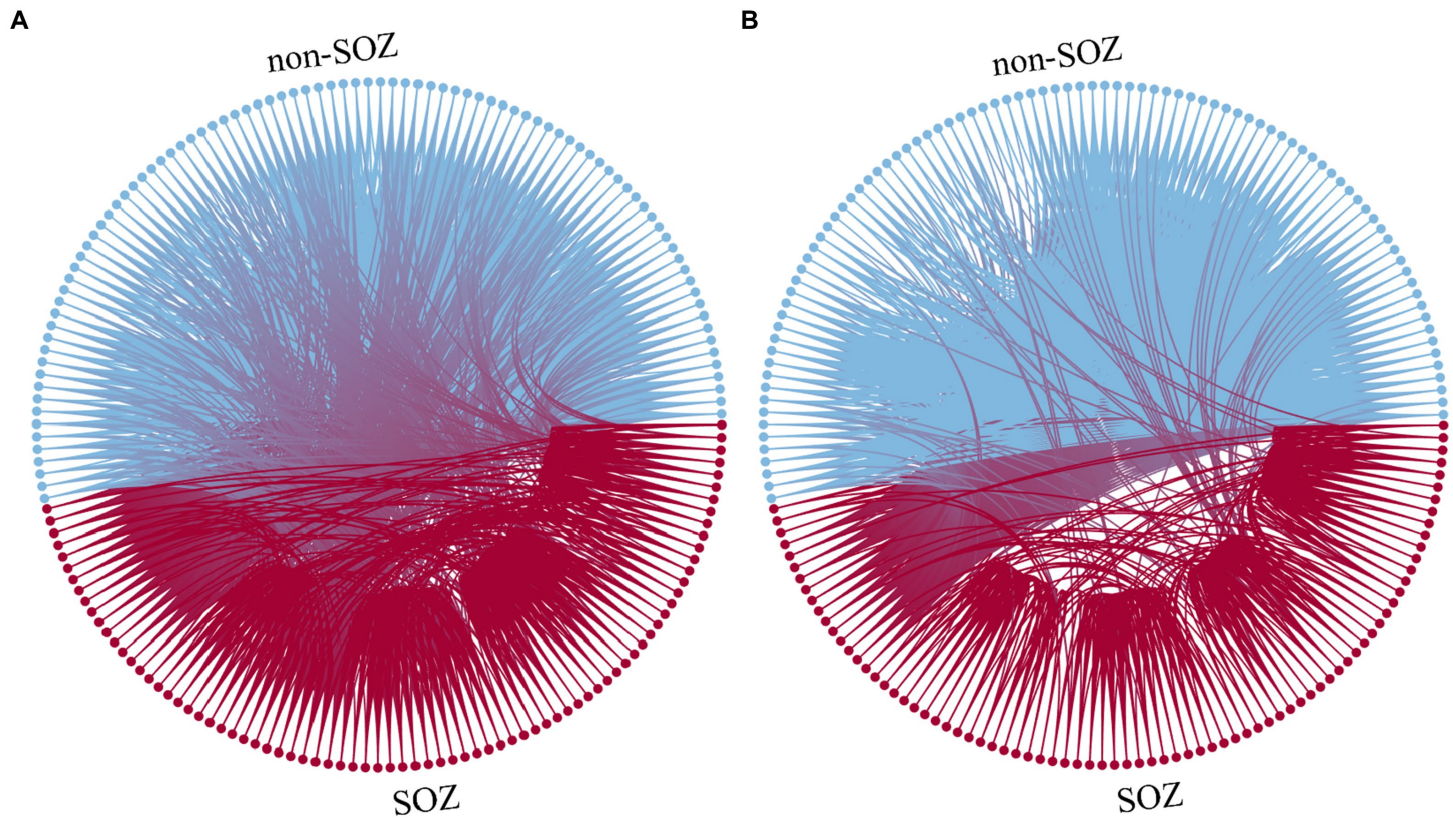
Function diagram of different staging. **(A)** Interictal. **(B)** Ictal. Blue: electrodes and connections in the non-SOZ region. Red: electrodes and connections in the SOZ region.

For instance, in the case of patient sub-HUP142, Figure 3 illustrates the functional connections of the first epoch at various stages in the gamma band.

**Figure 4:**
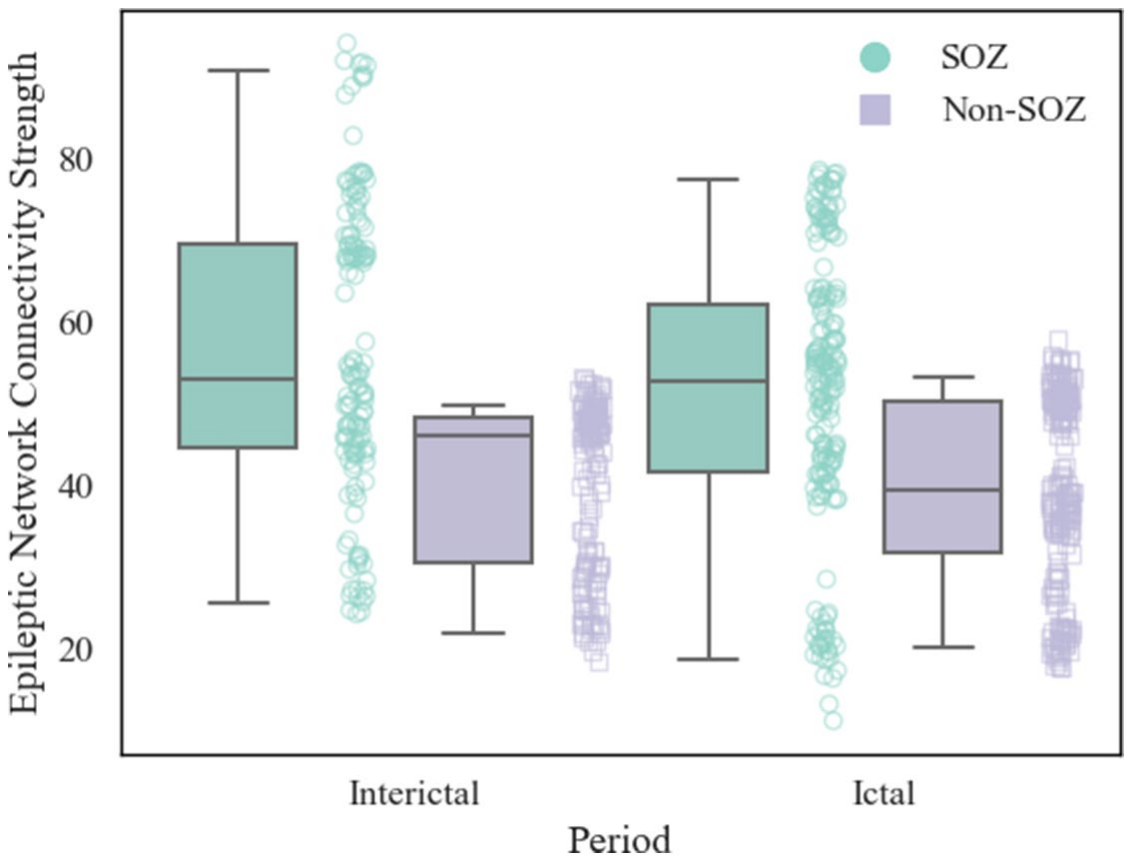
Comparison of ENCS between SOZ and non-SOZ area during different periods. The left side shows interictal data and the right side shows ictal data. Where green box plots: SOZ region ENCS data set; purple box plots: non-SOZ region ENCS data set; green round dots: corresponding to SOZ region ENCS data points; purple square dots: corresponding to non-SOZ region ENCS data points.

After constructing the brain functional networks, the mean ENCS of the brain functional networks in SOZ and non-SOZ areas of each sample within the gamma frequency band were computed. Subsequently, paired t-tests were conducted, with the results presented in Table 2. Our findings revealed significant differences at the 0.01 level in the ENCS of the brain functional networks between the SOZ and non-SOZ areas during both the ictal and interictal periods (*p* = 0.002 for the ictal period and *p* = 0.004 for the interictal period). In a specific comparison of the differences, it was observed that the mean value of ENCS of SOZ area nodes during the ictal period (51.40) was significantly higher than that of the nodes in the non-SOZ area, with ENCS mean value of 39.42. Similarly, during the interictal period, the mean ENCS value of SOZ nodes (56.66) was significantly higher compared to non-SOZ nodes, with an ENCS mean value of 40.06, as depicted in Figure 4. These findings suggest the presence of abnormal functional enhancement between neurons or neuronal populations within the SOZ area. Such pathological state may lead to structural and connectivity remodelling in the brain network, thereby resulting in an increase in the ENCS within the focal area. This remodelling process could be associated with pathological phenomena such as neuronal proliferation, neuronal migration, and so on.

**Table 2:** *T*-test Results for Different Regions of ENCS.

Periods	ENCS of SOZ	ENCS of non-SOZ	*p*
Ictal	51.40 ± 17.60	39.42 ± 11.75	0.002^**^
Interictal	56.66 ± 19.11	40.06 ± 10.47	0.004^**^

^*^p < 0.05 and ^**^p < 0.01.

### 3.2 Topological feature analysis

During the experiments, we employed the AMI method to determine the optimal delay time, along with the FNN algorithm to select the embedding dimension. Subsequently, we conducted delay time embedding for each channel, transforming the preprocessed EEG data into point cloud data.

**Figure 5:**
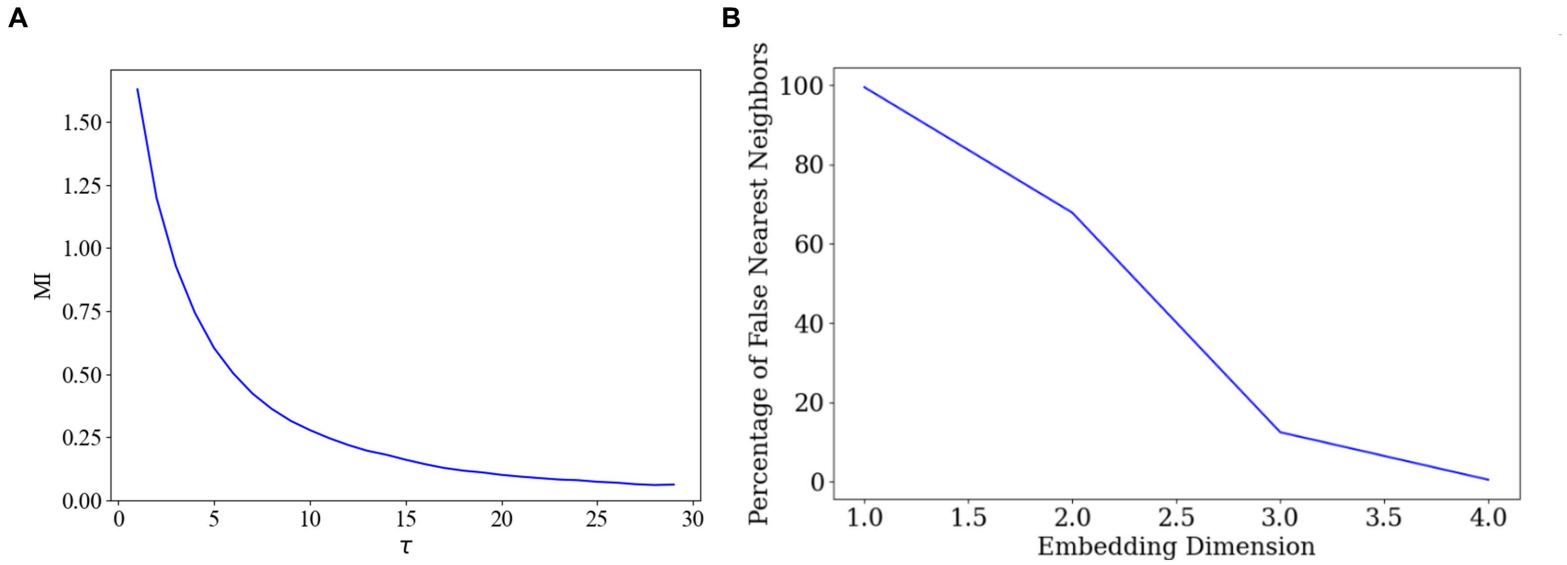
Optimal delay time and embedding dimension selection. **(A)** Determination of the delay time parameter. **(B)** Determination of the embedding dimensions.

First, we employed the AMI method to identify the optimal delay time. AMI is a widely used method for determining the optimal delay time in time-series data, aiming to maximise the preservation of the dynamic features of the original data during delayed embedding (Fraser and Swinney, 1986). By calculating the mutual information across various delay times and identifying the delay time corresponding to the peak mutual information, we could pinpoint the most appropriate delay time parameter 28, as depicted in Figure 5A.

Subsequently, we employed the FNN algorithm to determine the appropriate embedding dimensions. The FNN algorithm is commonly used for determining the appropriate dimensions in delay time embeddings, ensuring that the embedded data accurately represents the topology of the original data (Theiler et al., 1992). By comparing the distances between the original data points and their nearest and second nearest neighbours in the high-dimensional space, the algorithm identifies the dimension that minimizes the proportion of false nearest neighbours, thus determining the final embedding dimension 4, as illustrated in Figure 5B.

**Figure 6:**
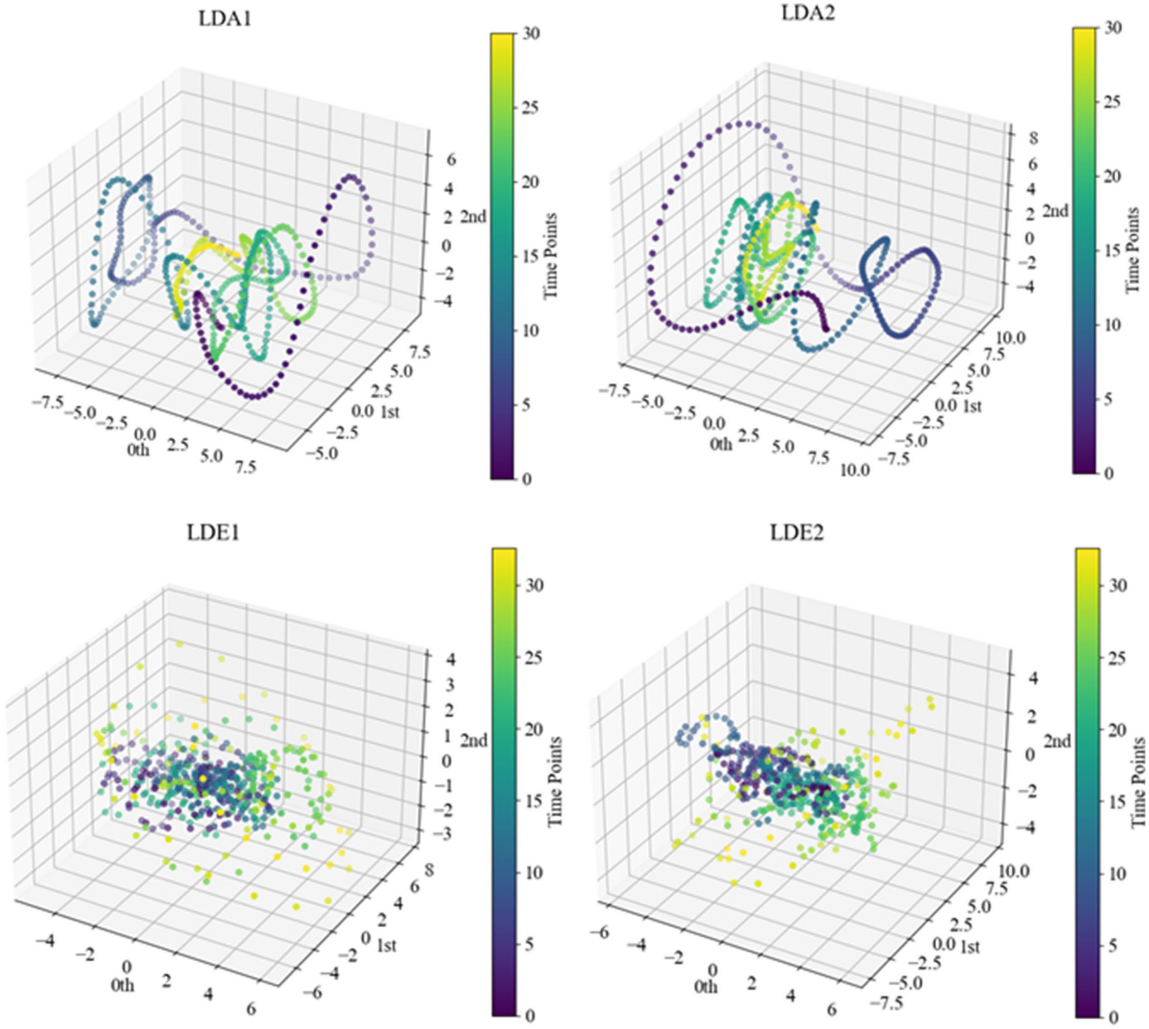
Point cloud data representation of some channels of sub-HUP142. The colors indicate the temporal information, i.e., the time sequence from the beginning to the end of the signals in this segment. LDA, left lateral temporal lobe; LDE, left occipital lobe (from the naming of the data set electrodes); LDA1 and LDA2, 2 SOZ area electrodes in the lateral part of the left temporal lobe; LDE1 and LDE2, 2 non-SOZ area electrodes in the left occipital lobe.

After determining specific delay times and embedding dimensions, we proceeded with delayed embedding of sub-HUP142 in the first 5 s window of each channel in the interictal SEEG signals based on the selected parameters. This process obtaining the corresponding point cloud data with a clearer topological structure. Then we visualised these embedded data in 3D space using principal component analysis (PCA), as shown in Figure 6. Within the same time window, the interictal point cloud data from epileptic focal zone channels exhibited more diverse array of attractor models in phase space. The morphology of these models demonstrated certain regularities, likely attributed to the interplay of local neuronal activity instability caused by pathological neural activity, the reorganisation of neuronal networks, and nonlinear dynamical properties. This observed regularity reflects the nonlinear dynamical properties within the brain during the epileptic state. Even during the interictal period, the brain activity exhibits a certain rhythmicity, which holds significance for understanding the pathogenesis and pathophysiological processes of epilepsy.

**Figure 7:**
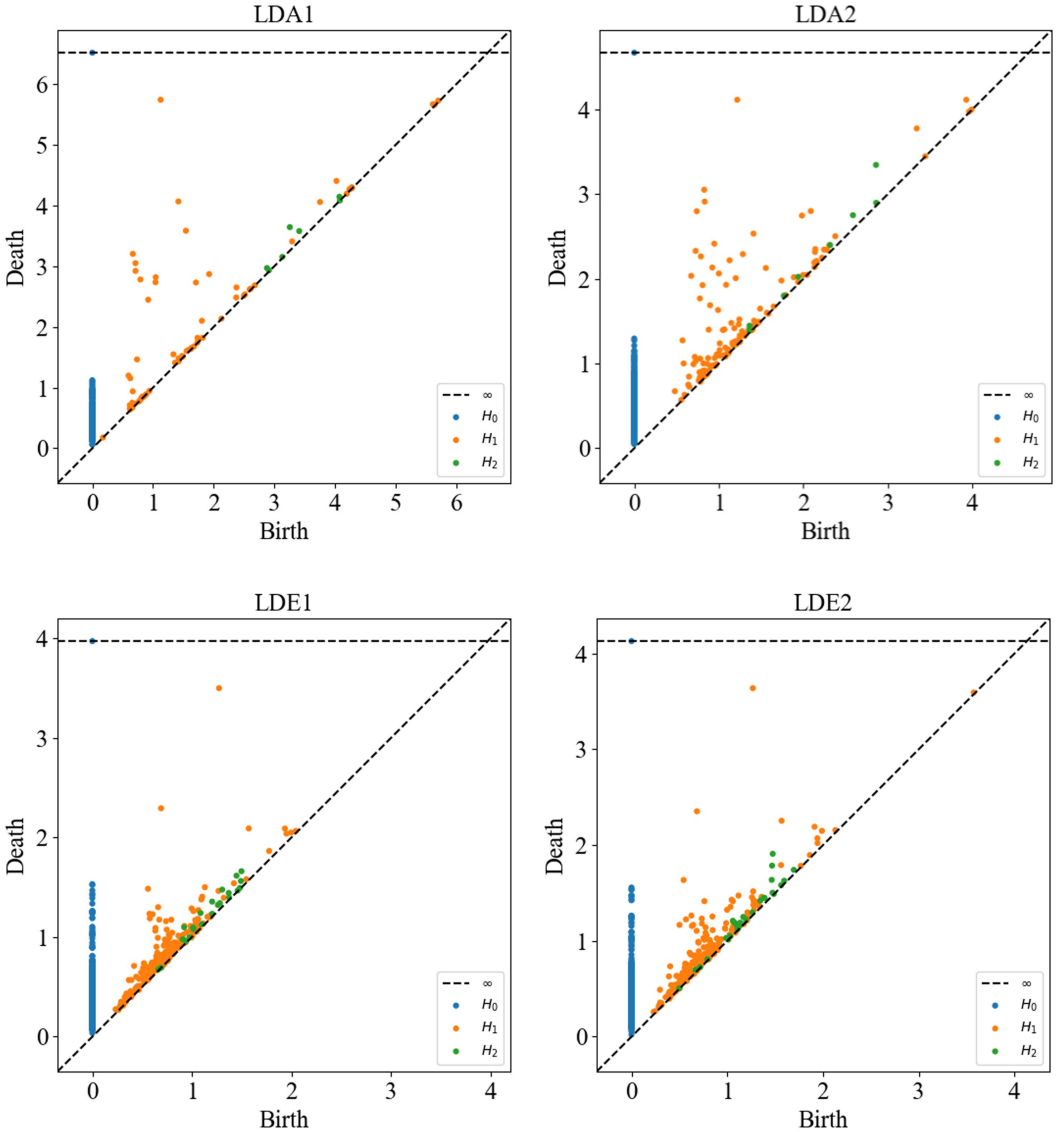
Persistent homology scatter plots of sub-HUP142 partial channels. H0: connected components (zero-dimensional homology group); H1: rings (one-dimensional homology group); H2: cavities (two-dimensional homology group). Each point represents one homology class. LDA, left lateral temporal lobe; LDE, left occipital lobe (from the naming of the data set electrodes); LDA1 and LDA2, 2 SOZ area electrodes in the lateral part of the left temporal lobe; LDE1 and LDE2, 2 non-SOZ area electrodes in the left occipital lobe.

Following this, Vietoris–Rips complex shape construction was performed on the point cloud data of each channel to compute the persistent homology maps. In Figure 7, the persistent homology scatter plots computed after Vietoris–Rips complex shape construction of some channels are presented. These plots contain cohomology information in different dimensions, namely H_0_, H_1_ and H_2_, respectively. Each point in H_0_ represents the survival interval of a connectivity component, i.e., the range of zero-dimensional holes, and may depict more connectivity components in non-SOZ areas. This could be attributed to the formation of more connections by neuronal activity in normal areas compared to SOZ areas, where broken connections or neuronal death may lead to fewer connectivity components. Each point in H_1_ represents the survival interval of a ring, i.e., the range of one-dimensional holes, while each point in H_2_ represents the survival interval of a hole, i.e., the range of two-dimensional holes. In non-SOZ areas, more complex and denser ring and hole structures may exist, possibly due to the formation of more loops and spatial structures by neuronal activity in normal areas. In contrast, SOZ areas may exhibit relatively lower densities of H_1_ and H_2_ due to abnormal neuronal activity leading to disruption or thinning of loops and holes.

**Figure 8:**
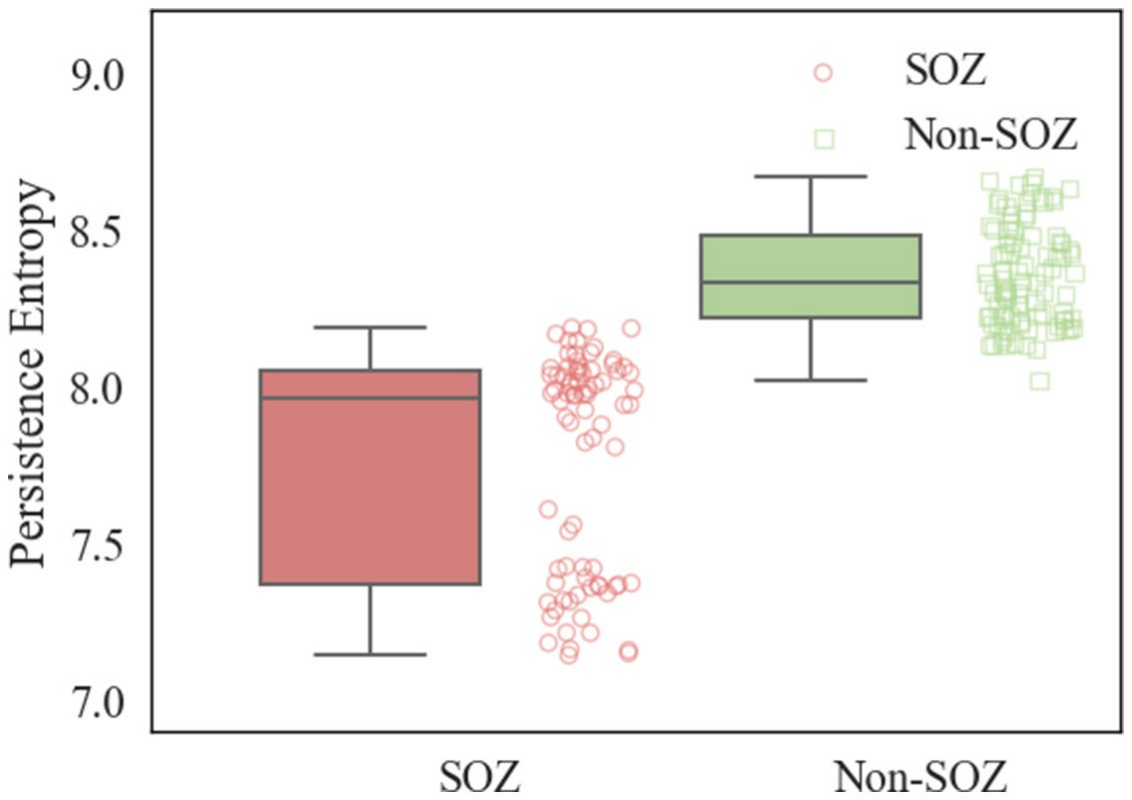
Comparison of PE in SOZ and non-SOZ area. Red box plots: SOZ region PE data sets; green box plots: non-SOZ region PE data sets; red round dots: correspond to SOZ region PE data points; green proto dots: correspond to non-SOZ region PE data points.

After constructing the persistent homology maps, the persistence entropy of the SOZ area and non-SOZ in the first 30 s of the interictal period of each sample was calculated, followed by paired t-tests for comparison. The results indicated that during the interictal period, significant differences in persistence entropy between the SOZ and non-SOZ areas at the 0.01 level (*p* < 0.01). Upon closer examination, it was found that the mean persistence entropy of the SOZ area nodes during the interictal period (7.77) was significantly lower than that of the non-SOZ area nodes (8.36), as depicted in Figure 8. This suggests that although the patient may be in a relatively stable state during the interictal period, some abnormal activities still exist at the neural network level. In this case, the lower persistence entropy in the lesion area may indicate a stronger ability of the neural network in this region to synchronise and regulate during the interictal period. This could be attributed to the relatively stable neuronal activity in the lesion area during the interictal period, which is less prone to sudden abnormal firing activity. This stable neural network state may contribute to a reduction in the randomness and chaos of information transmission, leading to a decrease in the persistence entropy. In contrast, the higher persistence entropy in the non-SOZ region may reflect the greater complexity and instability of the neural network in that region. During the interictal period, neuronal activity in the non-SOZ area may exhibit more heterogeneity and dynamics, making it susceptible to sudden changes in response to external factors. This instability of the neural network may result in increased stochasticity in information transmission, leading to relatively high persistence entropy.

### 3.3 Localisation of epileptic SOZ

In this study, SVM is employed for localisation of SOZ, with the where the RBF kernel function selected and a penalty parameter set to 0.5. Considering the significant individual variability among patients, we adopt an individualised training approach for each patient during the SOZ localisation experiments. Throughout the training phase, the data samples were partitioned into five subsample sets, ensuring an equal proportion of label composition for each category. Four of these sets were utilised for training purposes, while the remaining set was reserved for testing the model for 5-fold cross-validation. According to Supplementary Table S1, we chose to use the gamma band. The resultant localisation outcomes using ENCS for different patient localisation are detailed in Table 3. Subsequently, we introduce persistence entropy and integrate it with ENCS through feature fusion to enhance the localisation results for different patients, as illustrated in Table 4.

**Table 3:** Performance evaluation of localization model using ENCS (%).

Patient ID	Epoch	Positives	Negatives	AC	P	R	*F*1	AUC
sub-HUP117	89	50	39	88.89	98.18	80.92	87.19	91.04
sub-HUP130	224	108	116	97.78	99.05	96.51	97.73	98.93
sub-HUP133	133	63	70	94.76	100.00	89.64	94.36	98.58
sub-HUP138	198	100	98	97.97	100.00	95.96	97.87	99.16
sub-HUP142	172	80	92	88.35	85.80	91.33	87.84	94.71
sub-HUP144	171	75	96	91.24	100.00	80.20	88.78	95.78
sub-HUP150	161	81	80	94.41	100.00	88.93	93.93	94.40
sub-HUP151	332	168	164	97.58	100.00	95.01	97.38	98.28
sub-HUP157	292	144	148	95.54	100.00	90.88	95.20	99.12
sub-HUP160	202	125	77	88.12	97.03	82.86	89.32	93.27
sub-HUP162	312	156	156	95.19	98.59	91.84	95.03	98.03
sub-HUP164	212	45	167	94.35	94.11	77.95	84.77	91.15
sub-HUP166	244	100	144	91.00	97.40	80.71	87.80	93.84
sub-HUP171	252	108	144	98.42	100.00	96.07	97.98	99.41
sub-HUP172	246	125	121	97.58	99.00	95.60	97.20	98.50
sub-HUP173	194	95	99	87.60	89.63	84.01	86.72	95.65
sub-HUP180	209	105	104	95.23	97.35	93.45	95.21	99.48
sub-HUP181	262	132	130	97.33	99.33	95.21	97.17	99.65
sub-HUP185	212	108	104	96.70	100.00	93.17	96.36	98.53
sub-HUP187	143	70	73	90.22	100.00	80.84	89.19	96.38
sub-HUP188	273	140	133	88.27	96.27	79.85	87.17	91.74
sub-HUP190	245	120	125	91.43	100.00	82.90	90.39	99.27
Avg	—	—	—	93.54	97.81	88.36	92.48	96.59

**Table 4:** Performance evaluation of localization model using ENCS and PE (%).

Patient ID	Epoch	Positives	Negatives	AC	P	R	F1	AUC
sub-HUP117	89	50	39	89.93	96.67	82.58	88.99	90.94
sub-HUP130	224	108	116	97.78	99.13	96.23	97.61	98.48
sub-HUP133	133	63	70	95.53	100.00	91.64	95.42	98.47
sub-HUP138	198	100	98	97.97	100.00	95.96	97.87	99.16
sub-HUP142	172	80	92	94.81	91.32	98.95	94.93	99.14
sub-HUP144	171	75	96	91.24	100.00	80.20	88.78	97.17
sub-HUP150	161	81	80	94.41	100.00	88.93	93.93	96.50
sub-HUP151	332	168	164	97.58	100.00	95.01	97.38	98.10
sub-HUP157	292	144	148	94.86	100.00	89.35	94.33	98.79
sub-HUP160	202	125	77	87.63	98.89	85.14	88.70	93.17
sub-HUP162	312	156	156	95.51	99.26	91.84	95.35	97.85
sub-HUP164	212	45	167	95.34	96.67	81.11	85.80	91.51
sub-HUP166	244	100	144	91.84	100.00	80.60	88.53	94.63
sub-HUP171	252	108	144	98.42	100.00	96.07	97.98	99.47
sub-HUP172	246	125	121	97.58	99.00	95.60	97.20	98.98
sub-HUP173	194	95	99	93.31	96.45	90.44	93.07	95.83
sub-HUP180	209	105	104	95.23	97.35	93.45	95.21	99.35
sub-HUP181	262	132	130	97.33	99.33	95.21	97.17	99.62
sub-HUP185	212	108	104	96.70	100.00	93.17	96.36	98.45
sub-HUP187	143	70	73	93.77	94.83	89.89	92.10	97.39
sub-HUP188	273	140	133	88.64	97.76	80.12	87.60	93.78
sub-HUP190	245	120	125	91.43	100.00	82.90	90.39	96.54
Avg	—	—	—	94.40	98.48	89.74	93.40	96.97

**Figure 9:**
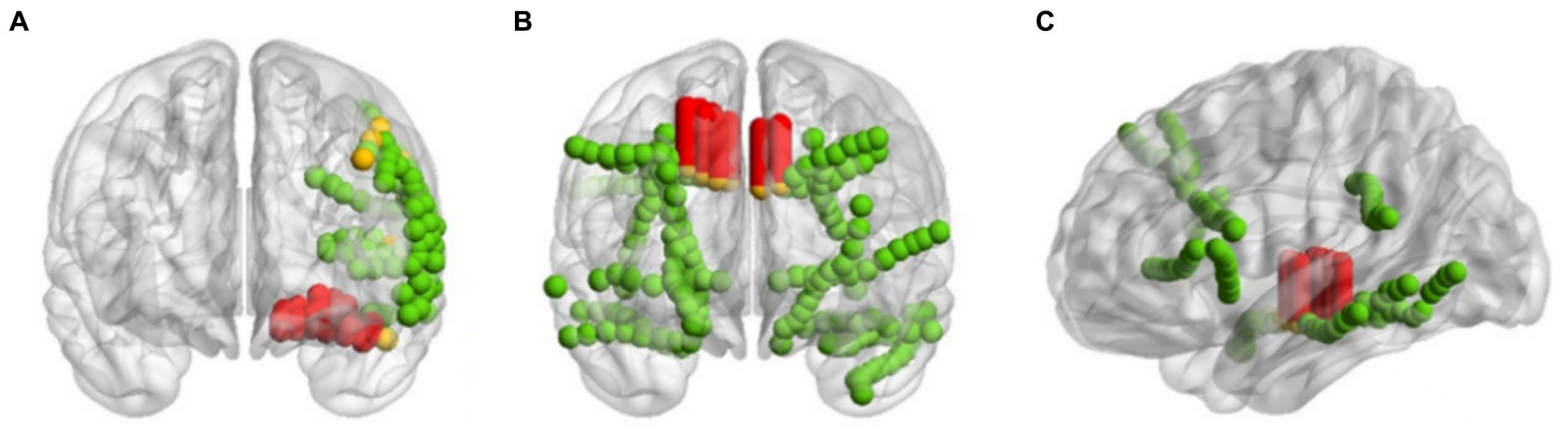
Imaging of positioning results. **(A)** sub-HUP142. **(B)** sub-HUP151. **(C)** sub-HUP181. Red solid circles: the electrodes that were correctly localised in epileptic SOZ; green solid circles: the electrodes that were correctly localised in non-SOZ; orange solid circles: the electrodes that were incorrectly localised.

Taking the three patients sub-HUP142, sub-HUP151 and sub-HUP181 as an example, as shown in Figure 9, the localised areas of epileptic SOZ for these patients are demonstrated. In the figure, the red solid circles represent the electrodes that were correctly localised in epileptic SOZ, the green solid circles indicate the electrodes that were correctly localised in non-SOZ, and the orange solid circles signify the electrodes that were incorrectly localised. Despite occasional mislocalisation, the overall results demonstrate superior localisation accuracy.

The research on localizing the SOZ using the HUP iEEG dataset is relatively limited, as indicated by Table 5, which presents the outcomes of experiments conducted on the same dataset. Among them, Sumsky and Santaniello (2018) explored EEG signals from 14 different patients (during 4 from the HUP iEEG dataset) sourced from the dataset of the National Institute of Neurological Disorders and Stroke iEEG portal (https://www.ieeg.org/, which contains the HUP iEEG dataset). They employed multichannel HFO rate in intracranial EEG as a feature to classify and identify SOZ using an SVM classifier, which achieved an accuracy of 0.92 ± 0.03 and an area under the ROC curve of 0.91 ± 0.03. Conrad et al. (2023) focused on using sleep-and seizure-associated spike changes to localise SOZ. They utilized a logistic regression classifier combined with spike rate to identify seizure zones, resulting in experimental outcomes with 92.1% accuracy and 44.3% precision. In summary, our proposed SOZ localisation method demonstrates slightly improved outcomes compared to similar studies. Specifically, it exhibits a 2.40 percentage point enhancement in localisation accuracy compared to Stefan et al. and an improvement of 2.30 percentage points in accuracy along with a substantial 54.18 percentage points in precision compared to Conrad et al.

**Table 5:** Comparison of related studies.

Author	Analysis method	Dataset	Classifier	Model performance		
				AC/%	P/%	AUC/%
Jiang et al.	Resting-state connectivity	A cohort of 27 drug-resistant focal epilepsy patients	RPC	88.00	—	94.00
Sumsky and Santaniello	HFO rate	HUP-iEEG dataset	SVM	92.00	—	91.00
Conrad et al.	Spike rate	HUP-iEEG dataset	LRC	92.10	44.30	82.00
Ours	WPLI	HUP-iEEG dataset	SVM	93.54	93.54	93.54
Ours	WPLI + PE	HUP-iEEG dataset	SVM	94.40	98.48	96.97

## 4 Discussion

In this research paper, brain networks assume an increasingly important role in the identification of epileptogenic SOZ and the prediction of post-surgical seizure outcome. Furthermore, SEEG, as a method for studying epilepsy, needs further comprehensive investigation. Presently, the identification of epileptogenic SOZ based on EEG signals has received garnered considerable attention, with high-frequency oscillatory and phase amplitude methods being predominant. However, these identification means focus on local signal features, ignoring the wholeness and complexity of brain networks. They often fail to take into account the interactions between nodes in network connections, as well as the nonlinear and nonstationary properties of neural signals. In recent years, there has been an increase in studies analyzing the relationship between interregional connectivity and the SOZ or epileptogenic zone, and these studies have contributed to our more comprehensive understanding of the neural mechanisms of epilepsy. Future studies should also pay more attention to the interactions between nodes in network connectivity, as well as the nonlinear and nonstationary properties of neural signals, so as to better reveal the pathophysiological processes of epilepsy.

In this study, we introduced the WPLI to construct brain functional networks for SOZ localisation. WPLI is widely utilized in EEG signals and has demonstrated promising results in epilepsy-related studies. For instance, Billeci et al. (2019) have utilized WPLI for feature extraction in a study of prediction of epileptic seizures, achieving an impressive average sensitivity of 93.3%, specificity of 80.6%, and prediction time of approximately 20 min. These outcomes underscore WPLI’s efficacy in assessing the interregional interactions or connectivity between brain regions. Hence, we believe that WPLI also holds considerable potential for localizing SOZ, and can also be effectively applied to SEEG signals, which can more accurately identify the abnormal connectivity patterns surrounding SOZ, thus furnishing more specific localisation information. In the analysis of the constructed brain functional networks, it was found that significant differences in the ENCS of the brain functional networks between SOZ and non-SOZ areas were revealed, laying a solid theoretical foundation for SOZ localization.

Currently, traditional EEG signal analysis methods have made significant strides in identifying brain regions involved in epileptic seizures. However, these methods are constrained by their low spatiotemporal resolution, rendering them insufficient for accurately localizing neural activity within the skull and limiting their clinical utility. In contrast, SEEG signals, acquired by intracranially implanted electrodes, offer higher spatial and temporal resolution, providing more precise spatial information. The superior spatial and temporal resolution of SEEG signals enables more accurate identification of abnormal connectivity patterns surrounding epileptic foci. Neuronal networks surrounding SOZ usually exhibit abnormal synchronisation and dysregulation, playing a pivotal role in seizure generation. Through analysis of SEEG signals, we can more precisely capture the characteristics of these abnormal connectivity pattern, thereby furnishing a more dependable foundation for SOZ localization. Recent studies (Xiao et al., 2021; Wang et al., 2023) have also demonstrated that SEEG shows potential in reflecting the distinction between epileptic and non-epileptic in brain networks and in identifying epileptic regions.

During the analysis of the constructed brain functional networks, significant differences were observed between the ENCS of the SOZ and non-SOZ areas during both interictal and ictal periods. Specifically, there was evident abnormal functional enhancement among neurons or neuronal populations within the focal areas. This pathological condition may contribute to the remodelling of brain network structures and connections, consequently leading to heightened ENCS within the focal areas. These findings provide a substantial theoretical basis for localizing the SOZ. In this study, we also delved into the dynamic properties of neural network activity during the interictal period of epilepsy, quantifying them through the theory of persistent homology. Our findings unveiled non-SOZ areas exhibited more intricate and denser connectivity components, rings, and hole structures compared to SOZ areas. Conversely, focal areas displayed disrupted or sparse connections due to abnormal neuronal activity, resulting in relatively lower densities of H_0_, H_1_ and H_2_ in the persistence homotopy maps. Additionally, we observed a notable contrast in persistence entropy between SOZ and non-SOZ areas during the interictal period. The diminished persistence entropy in the SOZ area whereas the elevated persistence entropy in non-SOZ areas suggests a more intricate and adaptable neural network, capable of swift adaptation to varying environments and task demands. These insights offer valuable references for comprehending the pathogenesis and pathophysiological processes of epilepsy. Future research endeavors could further investigate the interplay between dynamic neural network changes and epileptic seizures, providing enhanced theoretical foundations and clinical guidance for epilepsy diagnosis and treatment.

In this study, we proposed a model based on the structural features of brain functional networks to localise SOZ, incorporating nonlinear kinetic theory. Our model achieved an average accuracy of 94.40% for SOZ localisation, with a precision of 98.48%, a recall of 89.74%, an *F*1 score of 93.40%, and an AUC of 96.97%. Previously, most of the studies on localisation of SOZ have predominantly focused on analysing high-frequency oscillations using the HFO rate, but it has been demonstrated that the HFO rate alone is not statistically superior to the spikes in localising the SOZ and lacks the necessary sensitivity to serve as a standalone biomarker for SOZ localisation (Roehri et al., 2018). Additionally, It has also been demonstrated that for individual analyses, a reduced correlation between removal of HFO-generating regions at the group level and seizure-free outcome is reduced and some patients become seizure-free without removal of most of the HFO-generating regions (Jacobs et al., 2018). Balaji and Parhi (2022) noted that the efficacy of graph-theoretic methodology and phase-amplitude coupling also performs well in SOZ localisation. These findings not only provide a solid foundation for our study but also provide empirical support for the exploration and validation of brain functional network features and nonlinear dynamics in electrode localisation for SOZ. Furthermore, the results of this study provide a more precise examination and validation of the potential of brain functional network features and nonlinear dynamics in localizing SOZ.

Currently, epilepsy is considered a disorder associated with brain networks (San-Juan and Rodríguez-Méndez, 2023). Surgical resection, local ablation, or neuromodulation of these core epileptic propagation regions may help to stop seizures in patients with refractory epilepsy (Park and Madsen, 2018; Narasimhan et al., 2020). Several approaches have been devised for analyzing epileptic networks through frequency or time-frequency analysis of EEG signals (Sip et al., 2021; Matarrese et al., 2023; Stergiadis et al., 2023), but the propagation and onset of epileptic activity remain to be fully explored. The approach proposed in this study, based on epileptic network connection strength, offers a novel perspective and tool for investigating seizure propagation. By delving into the variations of ENCS within brain networks, we can observe that the pathways of seizure propagation tend to align with specific neural pathways, usually associated with specific regions or structures in the brain. This identification and characterization of seizure propagation pathways enhance our understanding of how epileptic foci localize and disseminate in the brain, thereby offering valuable insights for clinical diagnosis and treatment of epilepsy. In addition, these findings provide new directions and ideas for further research in the field of neuroscience, shedding light on the intricate structure and function of brain networks. However, it’s important to acknowledge that the study of seizure propagation pathways is still in the preliminary stage, with many unexplored areas awaiting further investigation. Future studies can delve deeper into the mechanism of seizure propagation, elucidate the characteristics and pathways of various seizure types, and develop more effective methods and strategies for individualised treatment of epilepsy and precision medicine.

## 5 Conclusion

In summary, our study provides valuable insights into localizing the SOZ. Through analysing the ENCS and the topology of neural signals, we uncovered the underlying mechanisms of epileptic activity and enhanced the accuracy of epileptic SOZ localisation. In the future, further research in this area may facilitate the development of more effective epilepsy treatment strategies.

However, it’s important to acknowledge that our model still has certain limitations. Firstly, our study was validated based on a specific dataset only, making it more susceptible to individual differences. Further multi-dataset studies are necessary to evaluate its applicability across different epilepsy types and patient groups. sEEG itself has some limitations in that it does not provide brain-wide data. Because sEEG records within the patient’s scalp through only a few electrodes, it cannot capture the activity of the entire brain. This localization limits the ability of sEEG in analyzing brain networks, especially for studies involving functional connectivity and network structure throughout the brain. Secondly, the construction and analysis of the brain functional network require the selection of various parameters, which need to be further optimised and standardised. Additionally, while the model has shown promising results in localizing epileptic SOZ, there is still room for improvement in terms of sensitivity.

We also need to note that although advanced methods such as WPLI were used in this study to assess brain network connectivity, we realize that electrode spacing may have an impact on the results when discussing brain network connectivity. This potential problem reminds us of the need to carefully select and consider electrode layouts when performing brain network analysis to minimize the impact of potential bias due to electrode spacing. Future studies will further explore the effect of electrode spacing on connection strength and attempt to take measures to verify the robustness of the observed connection patterns. Such work will contribute to a more comprehensive understanding of the structural and functional properties of brain networks and provide more accurate methods of data analysis for neuroscience research.

Despite encountering challenges and limitations, our study presents a novel approach for localizing epileptic SOZ. The method based on brain functional networks holds promise for enhancing individualized diagnosis and treatment of epilepsy. Future research endeavors could involve training and validating the model on larger datasets encompassing diverse epilepsy types and patient characteristics to enhance its applicability and generalizability. Furthermore, expanding the range of brain functional network features to include other relevant factors could improve the accuracy and comprehensiveness of epileptic focal zone localization.

## Data availability statement

Publicly available datasets were analyzed in this study. This data can be found at: doi: 10.18112/openneuro.ds004100.v1.1.3.

## Author contributions

CF: Conceptualization, Data curation, Funding acquisition, Project administration, Supervision, Writing – original draft. XL: Data curation, Formal analysis, Investigation, Methodology, Validation, Visualization, Writing – original draft. MN: Data curation, Writing – review & editing. WJ: Data curation, Writing – review & editing. YH: Investigation, Writing – review & editing. AW: Validation, Writing – review & editing. JH: Validation, Writing – review & editing. MZ: Validation, Writing – review & editing.
