# Supplementary material for: Epilepsy lesion localization method based on brain function network
Source: Front Hum Neurosci. 2024 Jul 10;18:1431153. doi: 10.3389/fnhum.2024.1431153 (PMC11266299; doi:10.3389/fnhum.2024.1431153)
Supplement: Supplementary file 1 [file Data_Sheet_1.docx]

Supplementary Material

Epilepsy lesion localization method based on brain function network

Chunying Fang ^1*^, Xingyu Li ^1*^,Meng Na ^2^, Wenhao Jiang ^3^, Yuankun He ^1^, Aowei Wei ^1^, Jie Huang ^1^, Ming Zhou ^1^

*** Correspondence:**Xingyu Li
lixingyu6868@163.com
Chunying Fang
fcy3333@163.com

# Supplementary Figures and Tables

## Supplementary Figures


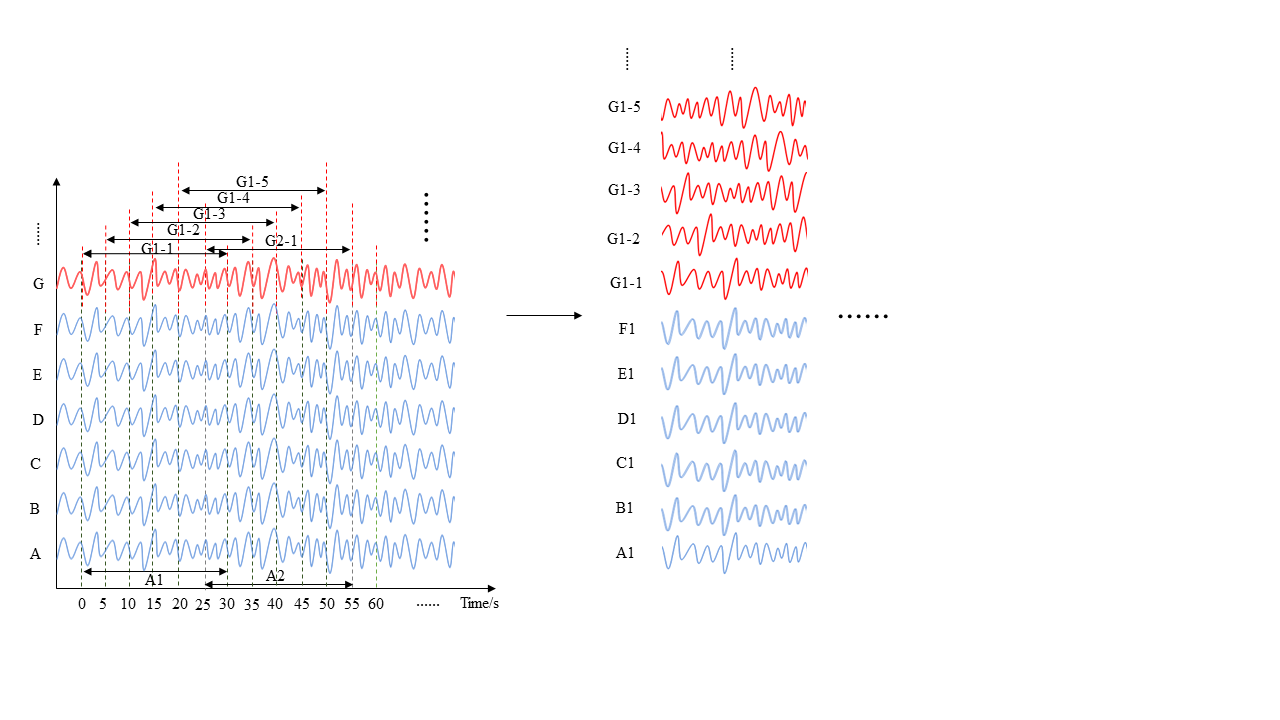


**Supplementary Figure 1.** Equalization Channel Abstraction Example Diagram. Blue line: the Non-SOZ EEG signals. Red line: the SOZ EEG signal.

Since the number of overall EEG signal channels is too large for a comprehensive display, we use abstract graphs for explanation and elaboration. As shown in the SUPPLEMENTARY FIGURE 1, the left figure shows the EEG signals before channel equalization, where the blue line represents the Non-SOZ EEG signals and the red line represents the SOZ EEG signals. The right figure shows the post channel equalization data, where the blue line represents the new Non-SOZ EEG signal and the red line represents the new SOZ EEG signal.

For the Non-SOZ channel EEG data, we used a window length of 30 seconds and a window shift of 25 seconds for slicing, for example, channel A. Meanwhile, the same operation was performed for other Non-SOZ channel EEG data, such as channels B to F. The same operation was performed for the lesion area channel EEG data. For SOZ channel EEG signal data, we used a 30-second window length, 5-second window shift for slicing, e.g., channel G. Meanwhile, the equivalent operation was also performed for other non-displayed SOZ channel EEG signal data.

Subsequently, we reorganized the Non-SOZ channel EEG signal slices such as A1 to F1 and SOZ channel EEG signal slices such as G1-1 to G1-5 into a new full-channel EEG signal data with a window length of 30 s. The same operation was performed on the slices of A2 to F2 and G2-1 to G2-5 to reorganize them into a second full-channel EEG signal data, and so on. And so on.

Taking sub-HUP142 as an example, the EEG data of this patient has 116 channels, of which 8 channels are bad channels. After removing the bad channels, there are 108 channels, 16 channels belonging to the SOZ region and 92 channels not belonging to the SOZ region, which is a very unbalanced sample. In this paper, we use the above method to expand the number of SOZ channel samples by 5 times, and after adjusting the sample equalization, the total number of channels (after removing the bad channels) becomes 172, and the number of SOZ channels becomes 80.

## Supplementary Tables

**Supplementary Table 1.** Accuracy of localization models based on different connectivity metrics(%).

| Patient ID | MI | Pearson | PLV | PLI | WPLI |
| --- | --- | --- | --- | --- | --- |
| sub-HUP117 | 80.15 | 86.76 | 86.76 | 85.39 | 87.65 |
| sub-HUP130 | 92.98 | 95.77 | 96.21 | 95.98 | 97.76 |
| sub-HUP133 | 94.74 | 91.23 | 92.74 | 87.22 | 91.74 |
| sub-HUP138 | 97.00 | 93.45 | 95.98 | 96.97 | 97.49 |
| sub-HUP142 | 94.09 | 91.02 | 88.70 | 76.16 | 93.03 |
| sub-HUP144 | 86.14 | 89.43 | 89.43 | 90.29 | 91.21 |
| sub-HUP150 | 90.79 | 92.41 | 92.41 | 92.55 | 93.79 |
| sub-HUP151 | 93.39 | 95.29 | 95.59 | 97.59 | 97.59 |
| sub-HUP157 | 90.15 | 92.52 | 92.52 | 93.84 | 95.58 |
| sub-HUP160 | 86.60 | 85.13 | 85.13 | 83.17 | 88.13 |
| sub-HUP162 | 92.19 | 93.83 | 93.83 | 94.87 | 96.48 |
| sub-HUP164 | 92.75 | 90.45 | 90.92 | 93.87 | 95.27 |
| sub-HUP166 | 86.34 | 89.80 | 89.80 | 91.39 | 91.81 |
| sub-HUP171 | 95.02 | 96.02 | 96.41 | 92.46 | 98.42 |
| sub-HUP172 | 93.34 | 94.75 | 95.97 | 97.56 | 97.55 |
| sub-HUP173 | 87.21 | 88.21 | 87.69 | 88.66 | 90.73 |
| sub-HUP180 | 90.78 | 94.65 | 94.65 | 94.26 | 96.66 |
| sub-HUP181 | 93.95 | 95.33 | 95.33 | 95.80 | 96.94 |
| sub-HUP185 | 92.75 | 91.87 | 93.75 | 95.28 | 96.69 |
| sub-HUP187 | 90.01 | 88.21 | 88.21 | 87.41 | 90.2 |
| sub-HUP188 | 86.01 | 87.74 | 87.74 | 86.45 | 89.02 |
| sub-HUP190 | 85.98 | 88.20 | 88.20 | 90.20 | 90.61 |
| Avg | 90.56 | 91.46 | 91.73 | 91.24 | 93.83 |

**Supplementary Table 2.** Precision of localization models based on different connectivity metrics(%).

| Patient ID | MI | Pearson | PLV | PLI | WPLI |
| --- | --- | --- | --- | --- | --- |
| sub-HUP117 | 79.90 | 87.80 | 87.80 | 85.64 | 97.14 |
| sub-HUP130 | 93.40 | 95.84 | 96.33 | 95.99 | 98.95 |
| sub-HUP133 | 94.73 | 91.61 | 93.45 | 87.17 | 93.7 |
| sub-HUP138 | 97.00 | 93.46 | 96.04 | 97.03 | 98.89 |
| sub-HUP142 | 94.25 | 91.89 | 90.59 | 76.07 | 88.08 |
| sub-HUP144 | 88.23 | 91.24 | 91.24 | 91.60 | 100 |
| sub-HUP150 | 91.02 | 92.94 | 92.94 | 92.77 | 98.46 |
| sub-HUP151 | 93.40 | 95.35 | 95.67 | 97.67 | 100 |
| sub-HUP157 | 90.24 | 93.12 | 93.12 | 94.29 | 100 |
| sub-HUP160 | 86.11 | 85.13 | 85.13 | 82.31 | 97.55 |
| sub-HUP162 | 92.28 | 94.15 | 94.15 | 95.06 | 99.39 |
| sub-HUP164 | 92.47 | 87.20 | 88.21 | 92.38 | 96 |
| sub-HUP166 | 86.93 | 91.90 | 91.90 | 93.64 | 100 |
| sub-HUP171 | 95.16 | 96.16 | 96.65 | 92.19 | 100 |
| sub-HUP172 | 93.34 | 94.75 | 96.02 | 97.59 | 99.23 |
| sub-HUP173 | 88.03 | 89.95 | 89.23 | 89.84 | 95.56 |
| sub-HUP180 | 90.82 | 94.85 | 94.85 | 94.26 | 98.29 |
| sub-HUP181 | 94.06 | 95.38 | 95.38 | 95.81 | 97.69 |
| sub-HUP185 | 92.75 | 91.93 | 94.02 | 95.48 | 100 |
| sub-HUP187 | 90.64 | 89.95 | 89.95 | 88.23 | 96.67 |
| sub-HUP188 | 87.32 | 89.30 | 89.30 | 87.79 | 98.18 |
| sub-HUP190 | 86.34 | 89.95 | 89.95 | 91.95 | 100 |
| Avg | 90.84 | 91.99 | 92.36 | 91.58 | 97.90 |

**Supplementary Table 3.** Recall of localization models based on different connectivity metrics(%).

| Patient ID | MI | Pearson | PLV | PLI | WPLI |
| --- | --- | --- | --- | --- | --- |
| sub-HUP117 | 80.31 | 88.00 | 88.00 | 86.15 | 80.73 |
| sub-HUP130 | 92.83 | 95.72 | 96.15 | 95.96 | 96.34 |
| sub-HUP133 | 94.86 | 91.02 | 92.44 | 87.22 | 89 |
| sub-HUP138 | 97.00 | 93.45 | 96.00 | 96.99 | 96.29 |
| sub-HUP142 | 93.96 | 90.58 | 88.00 | 76.17 | 97.49 |
| sub-HUP144 | 85.09 | 88.51 | 88.51 | 89.46 | 80.22 |
| sub-HUP150 | 90.81 | 92.44 | 92.44 | 92.57 | 88.71 |
| sub-HUP151 | 93.40 | 95.31 | 95.62 | 97.62 | 95.17 |
| sub-HUP157 | 90.12 | 92.44 | 92.44 | 93.77 | 91.13 |
| sub-HUP160 | 88.35 | 87.35 | 87.35 | 83.91 | 83.13 |
| sub-HUP162 | 92.19 | 93.83 | 93.83 | 94.87 | 93.86 |
| sub-HUP164 | 88.62 | 85.90 | 86.20 | 88.80 | 80.67 |
| sub-HUP166 | 85.07 | 88.00 | 88.00 | 89.50 | 79.99 |
| sub-HUP171 | 94.80 | 95.80 | 96.15 | 92.94 | 96.13 |
| sub-HUP172 | 93.35 | 94.76 | 96.00 | 97.59 | 95.95 |
| sub-HUP173 | 87.06 | 88.00 | 87.49 | 88.48 | 85.85 |
| sub-HUP180 | 90.77 | 94.67 | 94.67 | 94.26 | 95.09 |
| sub-HUP181 | 93.93 | 95.34 | 95.34 | 95.80 | 96.16 |
| sub-HUP185 | 92.76 | 91.91 | 93.83 | 95.35 | 93.55 |
| sub-HUP187 | 89.89 | 88.00 | 88.00 | 87.26 | 83.08 |
| sub-HUP188 | 86.25 | 88.00 | 88.00 | 86.69 | 79.9 |
| sub-HUP190 | 85.88 | 88.00 | 88.00 | 90.00 | 81.16 |
| Avg | 90.33 | 91.23 | 91.48 | 90.97 | 89.07 |

**Supplementary Table 4.** F1 score of localization models based on different connectivity metrics(%).

| Patient ID | MI | Pearson | PLV | PLI | WPLI |
| --- | --- | --- | --- | --- | --- |
| sub-HUP117 | 80.01 | 86.76 | 86.76 | 85.36 | 87.87 |
| sub-HUP130 | 92.96 | 95.76 | 96.21 | 95.98 | 97.59 |
| sub-HUP133 | 94.74 | 91.18 | 92.68 | 87.19 | 91.25 |
| sub-HUP138 | 97.00 | 93.45 | 95.98 | 96.97 | 97.51 |
| sub-HUP142 | 94.07 | 90.91 | 88.44 | 76.10 | 92.38 |
| sub-HUP144 | 85.72 | 89.13 | 89.13 | 89.99 | 88.95 |
| sub-HUP150 | 90.78 | 92.40 | 92.40 | 92.54 | 93.26 |
| sub-HUP151 | 93.39 | 95.29 | 95.59 | 97.59 | 97.52 |
| sub-HUP157 | 90.14 | 92.49 | 92.49 | 93.81 | 95.18 |
| sub-HUP160 | 86.39 | 84.94 | 84.94 | 82.68 | 89.64 |
| sub-HUP162 | 92.19 | 93.83 | 93.83 | 94.87 | 96.48 |
| sub-HUP164 | 90.38 | 86.53 | 87.15 | 90.44 | 86.96 |
| sub-HUP166 | 85.75 | 89.20 | 89.20 | 90.74 | 88.82 |
| sub-HUP171 | 94.97 | 95.97 | 96.37 | 92.39 | 97.99 |
| sub-HUP172 | 93.34 | 94.75 | 95.97 | 97.56 | 97.54 |
| sub-HUP173 | 87.13 | 88.07 | 87.56 | 88.54 | 90.09 |
| sub-HUP180 | 90.78 | 94.65 | 94.65 | 94.26 | 96.6 |
| sub-HUP181 | 93.94 | 95.33 | 95.33 | 95.80 | 96.91 |
| sub-HUP185 | 92.75 | 91.87 | 93.75 | 95.28 | 96.54 |
| sub-HUP187 | 89.97 | 88.07 | 88.07 | 87.31 | 89.03 |
| sub-HUP188 | 85.96 | 87.68 | 87.68 | 86.37 | 88.1 |
| sub-HUP190 | 85.93 | 88.06 | 88.06 | 90.06 | 89.39 |
| Avg | 90.38 | 91.20 | 91.47 | 90.99 | 92.98 |

**Supplementary Table 5.** AUC of localization models based on different connectivity metrics(%).

| Patient ID | MI | Pearson | PLV | PLI | WPLI |
| --- | --- | --- | --- | --- | --- |
| sub-HUP117 | 86.15 | 82.31 | 82.62 | 79.44 | 92.22 |
| sub-HUP130 | 97.92 | 96.37 | 96.92 | 96.61 | 98.37 |
| sub-HUP133 | 99.73 | 88.44 | 90.25 | 88.57 | 97.38 |
| sub-HUP138 | 100.00 | 98.03 | 96.48 | 98.09 | 99.95 |
| sub-HUP142 | 99.80 | 89.01 | 82.00 | 86.79 | 98.53 |
| sub-HUP144 | 84.38 | 82.16 | 82.66 | 83.70 | 93.98 |
| sub-HUP150 | 94.41 | 89.83 | 89.88 | 90.11 | 92.78 |
| sub-HUP151 | 95.30 | 95.84 | 95.84 | 95.77 | 98.35 |
| sub-HUP157 | 94.77 | 89.49 | 89.74 | 97.46 | 99.3 |
| sub-HUP160 | 86.10 | 81.64 | 82.48 | 84.39 | 93.77 |
| sub-HUP162 | 95.18 | 92.76 | 93.07 | 93.84 | 98.36 |
| sub-HUP164 | 95.66 | 81.90 | 81.06 | 82.59 | 90.44 |
| sub-HUP166 | 83.76 | 83.28 | 82.80 | 82.71 | 94.14 |
| sub-HUP171 | 98.24 | 96.77 | 96.87 | 96.43 | 97.9 |
| sub-HUP172 | 99.68 | 96.31 | 96.19 | 97.99 | 98.66 |
| sub-HUP173 | 84.58 | 82.51 | 82.48 | 81.37 | 95.01 |
| sub-HUP180 | 96.85 | 93.73 | 93.66 | 96.59 | 98.94 |
| sub-HUP181 | 98.72 | 95.75 | 95.79 | 96.85 | 98.79 |
| sub-HUP185 | 99.31 | 91.76 | 92.41 | 93.05 | 98.33 |
| sub-HUP187 | 94.17 | 83.09 | 81.50 | 82.94 | 96.2 |
| sub-HUP188 | 82.73 | 80.80 | 82.70 | 80.29 | 91.2 |
| sub-HUP190 | 94.60 | 81.51 | 81.65 | 82.40 | 96.71 |
| Avg | 93.73 | 88.79 | 88.59 | 89.45 | 96.33 |

**Supplementary Table 6.** Comparison of Localization Accuracy in Various Frequency Bands(%).

| Patient ID | Delta | Theta | Alpha | Beta | Gamma | Best Bands |
| --- | --- | --- | --- | --- | --- | --- |
| sub-HUP117 | 79.65 | 86.98 | 85.76 | **90.09** | 87.65 | Beta |
| sub-HUP130 | 90.22 | 94.20 | 95.20 | 96.21 | **97.76** | Gamma |
| sub-HUP133 | 86.64 | 90.73 | 90.25 | 91.25 | **91.74** | Gamma |
| sub-HUP138 | 89.47 | 93.49 | 94.49 | 95.46 | **97.49** | Gamma |
| sub-HUP142 | 88.50 | 90.19 | 91.79 | **93.31** | 93.03 | Beta |
| sub-HUP144 | 85.56 | 87.21 | 89.39 | 90.39 | **91.21** | Gamma |
| sub-HUP150 | 86.43 | 87.93 | 91.41 | 92.39 | **93.79** | Gamma |
| sub-HUP151 | 89.29 | 93.29 | 93.98 | 95.59 | **97.59** | gamma |
| sub-HUP157 | 85.49 | 91.23 | 91.89 | 92.51 | **95.58** | Gamma |
| sub-HUP160 | 77.23 | 83.16 | 87.11 | 86.56 | **88.13** | Gamma |
| sub-HUP162 | 87.20 | 91.20 | 92.84 | 93.51 | **96.48** | Gamma |
| sub-HUP164 | 85.84 | 91.75 | 92.28 | 92.32 | **95.27** | Gamma |
| sub-HUP166 | 83.40 | 87.81 | 89.64 | 89.79 | **91.81** | Gamma |
| sub-HUP171 | 90.41 | 94.42 | 95.42 | 96.40 | **98.42** | Gamma |
| sub-HUP172 | 89.56 | 93.14 | 94.55 | 95.57 | **97.55** | Gamma |
| sub-HUP173 | 84.75 | 86.73 | 89.28 | 84.64 | **90.73** | Gamma |
| sub-HUP180 | 88.18 | 91.23 | 93.66 | 94.63 | **96.66** | Gamma |
| sub-HUP181 | 89.71 | 93.33 | 93.18 | 93.39 | **96.94** | Gamma |
| sub-HUP185 | 88.22 | 92.22 | 93.69 | 94.71 | **96.69** | Gamma |
| sub-HUP187 | 85.69 | 85.56 | 86.53 | 86.84 | **90.20** | Gamma |
| sub-HUP188 | 81.39 | 84.28 | 86.38 | 87.34 | **89.02** | Gamma |
| sub-HUP190 | 84.25 | 86.61 | 87.20 | 89.02 | **90.61** | Gamma |
| Avg | 86.23 | 89.85 | 91.18 | 91.91 | **93.83** | Gamma |

In the preprocessing stage, we have extracted the signal data of different frequency bands separately, such as delta band (1 Hz to 3 Hz), theta band (3 Hz to 7 Hz), alpha band (7 Hz to 13 Hz), beta band (13 Hz to 30 Hz), gamma band (30 Hz to 60 Hz). Then we use WPLI to construct the brain functional network for the signals in each band, and extract the ENCS to construct the feature matrix.After that, we use the svm classifier to train and test the features in each band, and the results are shown in the Supplementary Table 6 (Note that no resampling was performed in the preprocessing of this comparison experiment, so it is normal that the results will differ from those in the manuscript).

First, we noted significant differences in the responses of each patient in different frequency bands. This may be due to biological differences between patients, including differences in brain structure, neural network connectivity patterns, and neural activity patterns. Second, even in the same frequency band, different patients showed different localization effects. This may be influenced by a variety of factors, including the severity of the condition, the type of seizure, medical history, and the effect of medication. These factors may cause different patients to exhibit different EEG characteristics in the same frequency band, thus affecting the performance of the localization model. We noticed that two patients in our sample showed the best localization results in the beta frequency band, while others achieved the best localization results in the gamma frequency band. This difference may reflect the complexity and diversity of epilepsy. beta frequency bands are often associated with functions such as motor control and emotion regulation, and thus may exhibit stronger features of EEG activity in some patients. In other patients, the gamma frequency band may be more capable of capturing EEG signals associated with seizures and therefore show better localization. Summarizing the above discussion, we believe that it is reasonable to choose gamma band signals for further study. First, the gamma frequency band showed better localization effects in most patients, which may be related to its more direct reflection of seizure-related EEG features. Second, there is a close correlation between high-frequency signals and epileptic seizures, so in-depth study of gamma-band signals can help us better understand the pathogenesis and EEG characteristics of epilepsy.
